# Facial reduction for symmetry reduced semidefinite and doubly nonnegative programs

**DOI:** 10.1007/s10107-022-01890-9

**Published:** 2022-09-27

**Authors:** Hao Hu, Renata Sotirov, Henry Wolkowicz

**Affiliations:** 1 School of Mathematical and Statistical Sciences, Clemson University, Clemson, South Carolina 29634 USA; 2 Department of Econometrics and Operations Research, Tilburg University, 5000 Tilburg, LE The Netherlands; 3 Department of Combinatorics and Optimization Faculty of Mathematics, University of Waterloo, Waterloo, Ontario N2L 3G1 Canada

**Keywords:** Semidefinite programming, Group symmetry, Facial reduction, Quadratic assignment problem, Vertex separator problem, 90C22, 90C25

## Abstract

We consider both facial reduction, **FR**, and symmetry reduction, **SR**, techniques for semidefinite programming, **SDP**. We show that the two together fit surprisingly well in an alternating direction method of multipliers, **ADMM**, approach. In fact, this approach allows for simply adding on nonnegativity constraints, and solving the doubly nonnegative, **DNN** , relaxation of many classes of hard combinatorial problems. We also show that the singularity degree remains the same after **SR**, and that the **DNN** relaxations considered here have singularity degree one, that is reduced to zero after **FR**. The combination of **FR** and **SR** leads to a significant improvement in both numerical stability and running time for both the **ADMM** and interior point approaches. We test our method on various **DNN** relaxations of hard combinatorial problems including quadratic assignment problems with sizes of more than $n=500$. This translates to a semidefinite constraint of order 250, 000 and $625\times 10^8$ nonnegative constrained variables, before applying the reduction techniques.

## Introduction

We consider two reduction techniques, facial and symmetry reduction, for semidefinite programming, **SDP**. We see that the exposing vector approach for *facial reduction*, ***FR***, moves naturally onto the *symmetry reduction*, ***SR***. We show that the combination of the two reductions fits surprisingly well in an alternating direction method of multipliers, **ADMM**, approach. In fact, the combination of **FR** and **SR** makes possible solving the doubly nonnegative, **DNN** , relaxations of many classes of hard combinatorial problems by using **ADMM**. The combination of facial and symmetry reduction also leads to a significant improvement in both numerical stability and running time for both the **ADMM** and interior point approaches. We test our method on various **DNN** relaxations of hard combinatorial problems including quadratic assignment problems (**QAP**) with sizes of more than $n=500$, see Table 1. Note that the order of the symmetric matrix variable in the **SDP** relaxation of the **QAP** with $n=500$, before applying the reduction techniques, is 250, 000. This yields approximately $625\times 10^8$ nonnegatively constrained variables in the semidefinite constrained matrix of the original, not reduced, problem formulation.

Semidefinite programming can be viewed as an extension of linear programming where the nonnegative orthant is replaced by the cone of positive semidefinite matrices. Although there are many algorithms for solving semidefinite programs, they currently do not scale well and often do not provide high accuracy solutions. An early method for exploiting sparsity and reducing problem size was based on recognizing a chordal pattern in the matrices forming the **SDP**, see e.g., [21, 32], and the survey [64]. Another technique is that of symmetry reduction, a methodology, pioneered by Schrijver [52], that exploits symmetries in the data matrices that allows for the problem size to be reduced, often significantly. More details and surveys for **SR** are available in [2, 13]

Without loss of generality, we consider the case where the primal problem has a finite optimal value. Then for linear programming, strong duality holds for both the primal and the dual problems. But, this is *not* the case for **SDP**, where the primal and/or the dual can be unattained, and one can even have a positive *duality gap* between the primal and dual optimal values. The usual constraint qualification to guarantee strong duality is the Slater condition, strict feasibility. Failure of the Slater condition may lead to theoretical and numerical problems when solving the **SDP**. Facial reduction, **FR**, introduced by Borwein and Wolkowicz [5–7], addresses this issue by projecting the *minimal face* of the **SDP** into a lower dimensional space. The literature for the theory and applications for **FR** is large. For a recent survey and theses see [18, 46, 55].

An earlier work [39] combines partial **FR** and **SR** for solving sum of square (**SOS**) programs. In particular, Löfberg [39] applies a partial **FR** via monomial basis selection and shows how to perform a partial **SR** via identification of sign-symmetries to obtain block-diagonal **SOS** programs. Examples in [39] verify the efficiency of the combined approach for **SOS** programs. For the connection between **FR** and monomial basis selection see [18, 65].

In our paper, we assume that we know how to do **FR** and **SR** separately for the input **SDP** instance. Under this assumption, we show that it is possible to implement **FR** to the symmetry reduced **SDP**. The obtained reduced **SDP** is both facially reduced and symmetry reduced. And, it can be solved in a numerically stable manner by interior point methods. Moreover, the nonnegativity constraints can be added to the original **SDP**, and the resulting **DNN** relaxation can be solved efficiently, as the nonnegativities follow through, and are in fact simplified, to the reduced **SDP** program. Thus, in fact we solve the facially and symmetry reduced **DNN** relaxation using an alternating direction method of multipliers approach, **ADMM**. As a consequence, we are able to solve some huge **DNN** relaxations for highly symmetric instances of certain hard combinatorial problems, and we do so in a reasonable amount of time.

We include theoretical results on facial reduction, as well as on the singularity degree of both **SDP** and **DNN** relaxations. We present a view of **FR** for **DNN** from the ground set of the original hard combinatorial problem. The singularity degree indicates the importance of **FR** for splitting type methods. In particular we show that the singularity degree remains the same after **SR**, and that our applications all have singularity degree one, that get reduced to zero after **FR**.

### Outline

In Sect. 2 we provide the background on using substitutions to first obtain **FR** and then symmetry and block diagonal **SR**. In Sect. 3 we show how to apply **FR** to the symmetry reduced **SDP**, and we also provide conditions such that the obtained **SDP** is strictly feasible. In fact, we show that the nonnegativity constraints are essentially unchanged and that we have strict feasibility for the reduced **DNN** relaxation. The results that singularity degree does not change after **SR** are included as well, see Sect. 3.3. In Sect. 4 we show that the reduced **DNN** relaxation can be solved efficiently using an **ADMM** approach. In Sect. 5 we apply our result to two classes of problems: the quadratic assignment and graph partitioning problems. Concluding comments are in Sect. 6.

## Background

### Semidefinite programming

The *semidefinite program*, ***SDP***, in standard form is

2.1 $$\begin{aligned} p^*_{{\textbf {SDP}}\,} := \min \{ \langle C,X \rangle \;|\; {{{\mathcal {A}}}(X)}=b, \;\; X \succeq 0\}, \end{aligned}$$

where the linear transformation ${{\mathcal {A}}}:{\mathbb {S}}^n\rightarrow {{\mathbb {R}}^m\,}$ maps real $n\times n$ symmetric matrices to ${{\mathbb {R}}^m\,}$, and $X\succeq 0$ denotes positive semidefiniteness, i.e., $X\in {\mathbb {S}}_+^n$. The set ${\mathbb {S}}_+^n$ is the *positive semidefinite*, ***PSD***, *cone*. In the case of a *doubly nonnegative*, ***DNN***, relaxation, nonnegativity constraints, $X\ge 0$, are added to (2.1), i.e., we use the ***DNN***  *cone* denoted ${\textbf {DNN}}\cong {\textbf {DNN}}\,^n = {\mathbb {S}}_+^n\cap {\mathbb {R}}^{n\times n}_+$. Without loss of generality, we assume that $\underline{{{\mathcal {A}}}\,\,\text {is onto}}$. We let

2.2 $$\begin{aligned} ({{\mathcal {P}}} _F) \qquad {{\mathcal {F}}} _X:=\{X \succeq 0 \,|\, {{{\mathcal {A}}}(X)}=b \} \end{aligned}$$

denote the *feasibility problem* for this formulation with data ${{\mathcal {A}}},b, {\mathbb {S}}_+^n$ from (2.1). Note that the linear equality constraint is equivalent to

$$\begin{aligned} {{\mathcal {A}}}(X)= \left( \langle A_i,X \rangle \right) = (b_i)\in {{\mathbb {R}}^m\,}, \end{aligned}$$

for some $A_i\in {\mathbb {S}}^n, i=1,\ldots ,m$. The *adjoint* transformation ${{{\mathcal {A}}}^*:{{\mathbb {R}}^m\,}\rightarrow {\mathbb {S}}^n}$ is ${{{\mathcal {A}}}^*(y)}=\sum _{i=1}^m y_iA_i$.

#### Strict feasibility and facial reduction

The standard constraint qualification to guarantee strong duality^1^ for the primal **SDP** is the *Slater constraint qualification* (strict feasibility)

$$\begin{aligned} \exists {\hat{X}} : \, \, \, {{\mathcal {A}}}({\hat{X}})=b, \, \, {\hat{X}} \succ 0, \end{aligned}$$

where ${\hat{X}}\succ 0$ denotes positive definiteness, i.e., ${\hat{X}} \in {\mathbb {S}}^n_{++}$. For many problems where strict feasibility fails, one can exploit structure and facially reduce the problem to obtain strict feasibility, see e.g., [5, 6] for the theory and [7] for the *facial reduction algorithm*.^2^ A survey with various views of **FR** is given in [18]. Facial reduction means that there exists a full column rank matrix $V\in {\mathbb {R}}^{n\times r}, r<n$, and the corresponding adjoint of the linear transformation ${{\mathcal {V}}} :{\mathbb {S}}^n\rightarrow {\mathbb {S}}^r$ given in

$$\begin{aligned} {{{\mathcal {V}}} ^*(R) = VRV^T},\, R \in {\mathbb {S}}^r, \end{aligned}$$

such that the *substitution* $X={{\mathcal {V}}} ^*(R)$ results in the *equivalent, regularized, smaller dimensional*, problem

2.3 $$\begin{aligned} p^*_{{\textbf {SDP}}\,}= \min \{ \langle V^{T}CV,R \rangle \;|\; \langle V^{T}A_{i}V,R \rangle = b_{i}, \;\; i \in {{\mathcal {I}}}\subseteq \{1,\ldots ,m\},\;\; R \in {\mathbb {S}}^r_+\}.\nonumber \\\end{aligned}$$

Strict feasibility holds for (2.3). The cone $V{\mathbb {S}}^r_+V^T$ is the *minimal face of the* **SDP**^3^, i.e., the smallest face of ${\mathbb {S}}_+^n$ that contains the feasible set, ${{\mathcal {F}}} _X$. And

$$\begin{aligned} {{\,\mathrm{range}\,}}(V) = {{\,\mathrm{range}\,}}(X), \, \forall X \in {{\,\mathrm{{relint}}\,}}({{\mathcal {F}}} _X). \end{aligned}$$

If $U\in {\mathbb {R}}^{n\times n-r}$ with ${{\,\mathrm{range}\,}}(U)={{\,\mathrm{null}\,}}(V^T)$, then $W:=UU^T$ is an *exposing vector* for the minimal face, i.e.,

$$\begin{aligned} X \text { feasible } \implies WX = 0. \end{aligned}$$

Let ${{\mathcal {F}}} _R$ denote the feasible set for (2.3). We emphasize the following constant rank result for the **FR** substitution:

$$\begin{aligned} R\in {{\mathcal {F}}} _R, \, {{\,\mathrm{{rank}}\,}}(R)=r \iff X={{\mathcal {V}}} ^*(R) \in {{\mathcal {F}}} _X, \, {{\,\mathrm{{rank}}\,}}(X)=r. \end{aligned}$$

##### Remark 2.1

For a typical **FR** algorithm for finding the minimal face, at each iteration the dimension is strictly reduced, and at least one redundant linear constraint can be discarded, i.e., we need at most $\min \{m,n-1\}$ iterations, e.g., [18, 56, 55, Theorem 3.5.4].

Note that **FR** can also be considered in the original space using rotations. Each step of **FR** involves finding an exposing vector $W=UU^T$ to the minimal face. Without loss of generality, we can assume that the matrix $$Q=\begin{bmatrix} V&U\end{bmatrix}$$ is orthogonal. Then the **FR** that reduces the size of the problem $X={{\mathcal {V}}} ^*(R) = VRV^T$ can equivalently be considered as a rotation (orthogonal congruence):

$$\begin{aligned} X = \begin{bmatrix} V&U \end{bmatrix} \begin{bmatrix} R &{}\quad 0 \\ 0 &{}\quad 0 \end{bmatrix} \begin{bmatrix} V&U \end{bmatrix}^T, \quad \begin{bmatrix} R &{}\quad 0 \\ 0 &{}\quad 0 \end{bmatrix} = \begin{bmatrix} V&U \end{bmatrix}^T X \begin{bmatrix} V&\quad U \end{bmatrix}, \end{aligned}$$

i.e., after this rotation, we can discard zero blocks and reduce the size of the problem. We note that this can then be compared to the *Constrained Set Invariance Conditions* approach in [46], where a special projection is used to obtain the reduced problem. In addition, the approach in [46] performs the projections on the primal-dual problem thus maintaining the original optimal values of both. In contrast, we emphasize the importance of the primal problem as being the problem of interest. After **FR** we have a regularized *primal* problem (2.3) with optimal value the same as that of the original *primal* problem. In addition, the reduced program has the important property that the dual of the dual is the primal.

### Group invariance and symmetry reduction, **SR**

We now find a *substitution* using the adjoint linear transformation ${\tilde{\mathbf{{\mathcal {B}}} }}^*(x)$ from (2.11) below, that provides the **SR** in block diagonal form. We first look at the procedure for simplifying an **SDP** that is invariant under the action of a symmetry group. This approach was introduced by Schrijver [52]; see also the survey [2]. The appropriate algebra isomorphism follows from the Artin-Wedderburn theory [66]. A more general framework is given in the thesis [46]. More details can be found in e.g., [12, 22, 24, 62].

Let ${{\mathcal {G}}}$ be a nontrivial group of permutation matrices of size *n*. The *commutant,* $A_{{{\mathcal {G}}} }$, (or *centralizer ring*) of ${{\mathcal {G}}}$ is defined as the subspace

2.4 $$\begin{aligned} A_{{{\mathcal {G}}} } := \{ X \in {\mathbb {R}}^{n\times n} \;|\; PX=XP, \; \forall P \in {{\mathcal {G}}} \}. \end{aligned}$$

Thus, $A_{{{\mathcal {G}}} }$ is the set of matrices that are self-permutation-congruent for all $P\in {{\mathcal {G}}}$. An equivalent definition of the commutant is

$$\begin{aligned} A_{{{\mathcal {G}}} } := \{X \in {\mathbb {R}}^{n\times n} \; | \; {{{\mathcal {R}}} _{{{\mathcal {G}}} }}(X) = X\}, \end{aligned}$$

where

2.5 $$\begin{aligned} {{{\mathcal {R}}} _{{{\mathcal {G}}} }}(X) :=\frac{1}{|{{{\mathcal {G}}} }|} \sum _{P\in {{{\mathcal {G}}} }} PXP^T, \,\, X\in {\mathbb {R}}^{n\times n}, \end{aligned}$$

is called the *Reynolds operator* (or *group average*) of ${{{\mathcal {G}}} }$. The operator ${{{\mathcal {R}}} _{{{\mathcal {G}}} }}$ is the orthogonal projection onto the commutant. The commutant $A_{{{\mathcal {G}}} }$ is a *matrix* $*$-*algebra*, i.e., it is a set of matrices that is closed under addition, scalar multiplication, matrix multiplication, and taking transposes. One may obtain a basis for $A_{{{\mathcal {G}}} }$ from the orbits of the action of ${{\mathcal {G}}}$ on ordered pairs of vertices, where the *orbit* of $(u_{i},u_{j}) \in \{0,1\}^{n} \times \{0,1\}^{n}$ under the action of ${{\mathcal {G}}}$ is the set $\{ (Pu_{i},Pu_{j}) \;|\; P \in {{\mathcal {G}}} \}$, and $u_i\in {\mathbb {R}}^n$ is the *i*-th unit vector. In what follows, we denote

2.6 $$\begin{aligned} \text {basis for } A_{{{\mathcal {G}}} }: \{ B_1, \ldots , B_d \},\, B_i \in \{0,1\}^{n\times n},\, \forall i. \end{aligned}$$

Let $J \cong J_n$ (resp. $I \cong I_n$) denote the matrix of all ones (resp. the identity matrix) of appropriate size. The basis (2.6) forms a so-called *coherent configuration*.

#### Definition 2.2

(*coherent configuration*) A set of zero-one $n\times n$ matrices $\{B_1,\ldots , B_d\}$ is called a coherent configuration of rank *d* if it satisfies the following properties:

1. $\sum _{i \in {\mathcal {I}} } B_i = I$ for some ${\mathcal {I}} \subset \{1,\ldots ,d\}$, and $\sum _{i=1}^d B_i = J$;
2. $$B_i^\mathrm {T} \in \{B_1,\ldots , B_d\}$$ for $$i=1,\ldots ,d$$;
3. $$B_iB_j \in {{\,\mathrm{{span}}\,}}\{B_1,\ldots , B_d\}, \, \forall i,j\in \{1,\ldots ,d\}$$.

In what follows we obtain that the Reynolds operator maps the feasible set ${{\mathcal {F}}} _X$ of (2.1) into itself and keeps the objective value the same, i.e.,

$$\begin{aligned} X \in {{\mathcal {F}}} _X \implies {{{\mathcal {R}}} _{{{\mathcal {G}}} }}(X) \in {{\mathcal {F}}} _X \text { and } \langle C, {{{\mathcal {R}}} _{{{\mathcal {G}}} }}(X) \rangle = \langle C, X \rangle . \end{aligned}$$

One can restrict optimization of an **SDP** problem to feasible points in a matrix $*$-algebra that contains the data matrices of that problem, see e.g., [14, 23]. In particular, the following result is known.

#### Theorem 2.3

( [14], Theorem 4) Let $A_{{{\mathcal {G}}} }$ denote a matrix $*$-algebra that contains the data matrices of an **SDP** problem as well as the identity matrix. If the **SDP** problem has an optimal solution, then it has an optimal solution in $A_{{{\mathcal {G}}} }$.

#### Remark 2.4

In [14], the authors consider complex matrix $*$-algebras. However in most applications, including applications in this paper, the data matrices are real symmetric matrices and $A_{{{\mathcal {G}}} }$ has a real basis, see Definition 2.2. Thus, we consider here the special real case. The authors in [14] also prove that if $A_{{{\mathcal {G}}} }$ has a real basis, and the **SDP** has an optimal solution, then it has a real optimal solution in $A_{{{\mathcal {G}}} }$. Real matrix $*$-algebras are also considered in [15, 16, 43].

In addition, Theorem 2.3 holds for **DNN**, i.e., we can move any nonnegativity constraints on *X* added to the **SDP** in Theorem 2.3 to simple nonnegativity constraints on *x* in (2.7), see e.g., [12, Pg 5].

Therefore, we may restrict the feasible set of the optimization problem to its intersection with $A_{{{\mathcal {G}}} }$. In particular, we can use the basis matrices and assume that

2.7 $$\begin{aligned} X\in {{\mathcal {F}}} _X \cap A_{{{\mathcal {G}}} } \Leftrightarrow \left[ X = \sum _{i=1}^{d}x_{i}B_{i} =: {\mathbf{{\mathcal {B}}} ^*(x)} \in {{\mathcal {F}}} _X, \text { for some } x\in {\mathbb {R}}^d \right] . \end{aligned}$$

From now on we assume that ${{\mathcal {G}}}$ is such that $A_{{{\mathcal {G}}} }$ contains the data matrices of (2.1).

#### Example 2.5

(Hamming Graphs) We now present an example of an algebra that we use later in our numerics.

The *Hamming graph* *H*(*d*, *q*) is the Cartesian product of *d* copies of the complete graph $K_q$, with vertices represented by *d*-tuples of letters from an alphabet of size *q*. The Hamming distance between vertices *u* and *v*, denoted by |(*u*, *v*)|, is the number of positions in which *d*-tuples *u* and *v* differ.

The matrices

$$\begin{aligned} (B_{i})_{u,v} := {\left\{ \begin{array}{ll} 1 &{} \text { if } |(u,v)| = i \\ 0 &{} \text { otherwise} \end{array}\right. }, \quad i = 0,\ldots ,d \end{aligned}$$

form a basis of the Bose-Mesner algebra of the Hamming scheme, see [17]. In particular, $B_{0} = I$ is the identity matrix and $B_1$ is the adjacency matrix of the Hamming graph *H*(*d*, *q*) of size $q^d \times q^d$. In cases, like for the Bose-Mesner algebra, when one of the basis elements equals the identity matrix, it is common to set the index of the corresponding basis element to zero. The basis matrices $B_{i}$ can be simultaneously diagonalized by the real, orthogonal matrix *Q* given by

$$\begin{aligned} Q_{u,v} = 2^{-\frac{d}{2}}(-1)^{u^{T}v}. \end{aligned}$$

The distinct elements of the matrix $Q^{T}B_{i}Q$ equal $K_{i}(j)$ ($j=0,\ldots ,d$) where

$$\begin{aligned} K_{i}(j) := \sum _{h=0}^{i} (-1)^{h}(q-1)^{i-h}{j \atopwithdelims ()h} {d-j \atopwithdelims ()i-h}, \quad j = 0,\ldots ,d, \end{aligned}$$

are *Krawtchouk polynomials*. We denote by $\mu _j := {d \atopwithdelims ()j}(q-1)^{j}$ the multiplicity of the *j*-th eigenvalue $K_{i}(j)$. The elements of the character table $P \in {\mathbb {R}}^{(d+1) \times (d+1)}$ of the Hamming scheme *H*(*d*, *q*), given in terms of the Krawtchouk polynomials, are

$$\begin{aligned} p_{i,j} := K_{i}(j), \, i,j = 0,\ldots ,d. \end{aligned}$$

In the later sections, we use the following well-known orthogonality relations on the Krawtchouk polynomial, see e.g., [17],

2.8 $$\begin{aligned} \sum _{j=0}^{d}K_{r}(j)K_{s}(j) {d \atopwithdelims ()j}(q-1)^{j} = q^{d} {d \atopwithdelims ()s}(q-1)^{s} \delta _{r,s}, \quad r,s =0,\ldots ,d, \end{aligned}$$

where $\delta _{r,s}$ is the Kronecker delta function.

#### First symmetry reduction using $X=\mathbf{{\mathcal {B}}} ^*(x)$

We now obtain our first reduced program using the substitution $X=\mathbf{{\mathcal {B}}} ^*(x)$. Note that the program is reduced in the sense that the feasible set can be smaller though the optimal value remains the same.

2.9 $$\begin{aligned} p^*_{{\textbf {SDP}}\,}= \min \{ \langle \mathbf{{\mathcal {B}}} (C),x \rangle \;|\; ({{\mathcal {A}}}\circ {\mathbf{{\mathcal {B}}} ^*})(x)=b, \;\; {\mathbf{{\mathcal {B}}} ^*}(x) \succeq 0\}, \quad (\text {substitution } X=\mathbf{{\mathcal {B}}} ^*(x)).\nonumber \\ \end{aligned}$$

Here, $\mathbf{{\mathcal {B}}}$ is the adjoint of $\mathbf{{\mathcal {B}}} ^*$. In the case of a **DNN** relaxation, the structure of the basis in (2.6) allows us to equate $X=\mathbf{{\mathcal {B}}} ^*(x) \ge 0$ with the simpler $x\ge 0$. This changes the standard doubly nonnegative cone into a splitting, the Cartesian product of the cones $x\ge 0, {\mathbf{{\mathcal {B}}} ^*}(x)\succeq 0$, see  Remarks 2.4 and 5.1.

A matrix $*$-algebra ${\mathcal {M}}$ is called *basic* if ${\mathcal {M}} = \{ \oplus _{i=1}^{t} M \;|\; M \in {\mathbb {C}}^{m \times m} \}$, where $\oplus$ denotes the direct sum of matrices. A very important decomposition result for matrix $*$-algebras is the following result due to Wedderburn.

##### Theorem 2.6

([66]) Let ${\mathcal {M}}$ be a matrix $*$-algebra containing the identity matrix. Then there exists a unitary matrix *Q* such that $Q^{*}{\mathcal {M}}Q$ is a direct sum of basic matrix $*$-algebras.

The above result is derived for a complex matrix $*$-algebras. In [43], the authors study numerical algorithms for block-diagonalization of matrix $*$-algebras over ${\mathbb {R}}$. Unfortunately, the Wedderburn decomposition described in the above theorem does not directly apply for $*$-algebras over reals. To demonstrate our approach in the section on numerical results we use known orthogonal matrices or a simple heuristics to obtain them.

To simplify our presentation, the matrix *Q* in Theorem 2.6 is assumed to be real orthogonal. (The case when *Q* is complex can be derived analogously.) Then, the matrices in the basis $B_{j},\, j= 1,\ldots ,d$, can be mutually block-diagonalized by some orthogonal matrix *Q*. More precisely, there exists an orthogonal matrix *Q* such that we get the following block-diagonal transformation on $B_j$:

2.10 $$\begin{aligned} {{\tilde{B}} _j := Q^{T}B_{j}Q =: {{\,\mathrm{{Blkdiag}}\,}}( ({\tilde{B}}_j^k)_{k=1}^t)}, \forall j=1,\ldots ,d. \end{aligned}$$

For $Q^{T}XQ = \sum _{j=1}^{d}x_{j}{\tilde{B}}_{j}$, we now define the linear transformation for obtaining the block matrix diagonal form:

2.11 $$\begin{aligned} {{\tilde{\mathbf{{\mathcal {B}}} }}^*(x)} := \sum _{j=1}^{d}x_{j}{\tilde{B}}_{j} = \begin{bmatrix} {\tilde{\mathbf{{\mathcal {B}}} }}^*_{1}(x) &{} &{} \\ &{} \ddots &{} \\ &{} &{} {\tilde{\mathbf{{\mathcal {B}}} }}^*_{t}(x) \end{bmatrix} =: {{\,\mathrm{{Blkdiag}}\,}}(({\tilde{\mathbf{{\mathcal {B}}} }}^*_k(x))_{k=1}^t), \end{aligned}$$

where

$$\begin{aligned} {{\tilde{\mathbf{{\mathcal {B}}} }}^*_{k}}(x) =: \sum _{j=1}^d x_j{{\tilde{B}}^k_j} \in {\mathcal {S}}^{n_{i}}_+ \end{aligned}$$

is the *k*-th diagonal block of ${\tilde{\mathbf{{\mathcal {B}}} }}^*(x)$, and the sum of the *t* block sizes $n_{1}+ \ldots +n_{t} = n$. Thus, for any feasible *X* we get

$$\begin{aligned} X = {\mathbf{{\mathcal {B}}} ^*}(x) = Q{\tilde{\mathbf{{\mathcal {B}}} }}^*(x)Q^T \in {\mathcal {F}}_{X}. \end{aligned}$$

#### Second symmetry reduction to block diagonal form using $X=Q{\tilde{\mathbf{{\mathcal {B}}} }}^*(x)Q^T$

We now derive the second reduced program using the substitution $X=Q{\tilde{\mathbf{{\mathcal {B}}} }}^*(x)Q^T$. The program is further reduced since we obtain the block diagonal problem

2.12 $$\begin{aligned} p^*_{{\textbf {SDP}}\,}= \min \{ \langle {\tilde{\mathbf{{\mathcal {B}}} }}({{\tilde{C}}}),x \rangle \;|\; ({{{\tilde{{{\mathcal {A}}}}}}}\circ {\tilde{\mathbf{{\mathcal {B}}} }}^*)(x)=b, \;\; {\tilde{\mathbf{{\mathcal {B}}} }}^*(x) \succeq 0\}, \end{aligned}$$

where $${{\tilde{C}}} = Q^TCQ$$ and $${{{\tilde{{{\mathcal {A}}}}}}}$$ is the linear transformation obtained from $${{\mathcal {A}}}$$ as follows: $${{\tilde{A}}}_j = Q^TA_jQ, \forall j$$. We denote the corresponding blocks as $${{\tilde{A}}}_j^k$$$$, \forall j =1,\ldots ,d, \forall k = 1,\ldots , t$$.

We see that the objective in (2.12) satisfies

$$\begin{aligned} {{{\tilde{c}}} : = {\tilde{\mathbf{{\mathcal {B}}} }}({{\tilde{C}}})} = (\langle {{\tilde{B}}}_j, {{\tilde{C}}} \rangle ) = (\langle B_j, C \rangle )\in {\mathbb {R}}^d. \end{aligned}$$

While the *i*-th row of the linear equality constraint in (2.12), ${{\tilde{A}}} x = b$, is

$$\begin{aligned} \begin{array}{rcl} b_i &{}=&{} ({{\tilde{A}}}x)_i \\ {} &{}:=&{} (({{{\tilde{{{\mathcal {A}}}}}}}\circ {\tilde{\mathbf{{\mathcal {B}}} }}^*)(x))_i \\ {} &{}=&{} \langle {{\tilde{A}}}_i, {\tilde{\mathbf{{\mathcal {B}}} }}^*(x) \rangle \\ {} &{}=&{} \langle {\tilde{\mathbf{{\mathcal {B}}} }}({{\tilde{A}}}_i), x \rangle . \end{array} \end{aligned}$$

Therefore

2.13 $$\begin{aligned} {{\tilde{A}}}_{ij} = ({\tilde{\mathbf{{\mathcal {B}}} }}({{\tilde{A}}}_i))_j = \langle {{\tilde{B}}}_j,{{\tilde{A}}}_i\rangle = \langle B_j,A_i\rangle , \quad i=1, \ldots ,m, \, j=1,\ldots ,d. \nonumber \\ \end{aligned}$$

Without loss of generality, we can now define

$$\begin{aligned} c:={{\tilde{c}}}, \quad A := {{\tilde{A}}}. \end{aligned}$$

Moreover, just as for **FR**, the **SR** step can result in *A* not being full row rank (onto). We then have to choose a nice (well conditioned) submatrix that is full row rank and use the resulting subsystem of $Ax=b$. We see below how to do this and simultaneously obtain strict feasibility.

We can now rewrite the **SDP**  (2.1) as

2.14 $$\begin{aligned} p^*_{{\textbf {SDP}}\,}= \min \{ c^{T}x \;|\; {Ax=b}, \;\; {\tilde{\mathbf{{\mathcal {B}}} }}^*_{k}(x) \succeq 0, \, k = 1,\ldots ,t \}. \end{aligned}$$

For many applications, there are *repeated blocks*. We then take advantage of this to reduce the size of the problem and maintain stability.

The program (2.14) is a *symmetry reduced formulation* of (2.1). We denote its feasible set and feasible slacks as

2.15 $$\begin{aligned} {{\mathcal {F}}} _x := \{x \;|\; {\tilde{\mathbf{{\mathcal {B}}} }}^*(x)\succeq 0,\, Ax=b,\,x\in {\mathbb {R}}^d\},\quad {\mathcal {S}}_x:= \{{\tilde{\mathbf{{\mathcal {B}}} }}^*(x)\succeq 0\;|\; Ax=b,\,x\in {\mathbb {R}}^d\}.\nonumber \\ \end{aligned}$$

We denote the *feasibility problem* for this formulation with data ${\tilde{\mathbf{{\mathcal {B}}} }}^*,A,b, {\mathbb {S}}_+^n$ of the feasible set ${{\mathcal {F}}} _x$ as ${{\mathcal {P}}} _{F_x}$. We bear in mind that ${\tilde{\mathbf{{\mathcal {B}}} }}^*(x)$ is a block-diagonal matrix. But it is written as a single matrix for convenience in order to describe **FR** for the symmetry reduced program below.

Since ${\tilde{B}}_{1},\ldots ,{\tilde{B}}_{d}$ are block diagonal, symmetric matrices, the symmetry reduced formulation is typically much smaller than the original problem, i.e.,

$$\begin{aligned} x \in {\mathbb {R}}^d, \quad d \ll \sum _{i=1}^dt(n_i) \ll t(n), \end{aligned}$$

where $t(k)=k(k+1)/2$ is the triangular number.

## Facial reduction for the symmetry reduced program

In this section, we show how to apply **FR** to the symmetry reduced **SDP**  (2.14). The key is using the exposing vector view of facial reduction, [18]. Formally speaking, if an exposing vector of the *minimal face* of the **SDP**  (2.1) is given, then we are able to construct a corresponding exposing vector of the minimal face of the symmetry reduced program (2.14). In fact, we show that all the exposing vectors of the symmetry reduced program can be obtained from the exposing vectors of the original program. In general, one can find exposing vectors from the original program by exploiting the structure. However, this is lost after the **SR** and results in a more difficult task in finding an exposing vector.

In addition, we follow the above theme on simply adding on the nonnegativities and extend many of the results to the **DNN** program. We include results on the singularity degree to emphasize the importance of **FR** for stability and that **SR** does *not* increase the singularity degree.

### Rank preserving

We begin with showing the maximum rank preserving properties of **SR**. Note that

$$\begin{aligned} \begin{array}{rcl} \max \{{{\,\mathrm{{rank}}\,}}(X) \,|\, X \in {{\mathcal {F}}} _X \} &{}=&{} {{\,\mathrm{{rank}}\,}}(X), \,\, \forall X \in {{\,\mathrm{{relint}}\,}}({{\mathcal {F}}} _X) \\ {} &{}=&{} {{\,\mathrm{{rank}}\,}}(X), \,\, \forall X \in {{\,\mathrm{{relint}}\,}}({{\,\mathrm{face}\,}}({{\mathcal {F}}} _X)), \end{array} \end{aligned}$$

where ${{\,\mathrm{face}\,}}({{\mathcal {F}}} _X)$ is the minimal face of ${\mathbb {S}}_+^n$ containing the feasible set. We let $F \unlhd K$ denote that *F* is a face of the cone *K*.

#### Theorem 3.1

Let $$r = \max \{ {{\,\mathrm{{rank}}\,}}(X) \,|\, X\in {{\mathcal {F}}} _X \}.$$ Then

$$\begin{aligned} \begin{array}{rcl} r&{}=&{} \max \left\{ {{\,\mathrm{{rank}}\,}}\left( {\frac{1}{|{{\mathcal {G}}} |}\sum _{P \in {{\mathcal {G}}} }P^{T}XP} \right) \, | \, X \in {{\mathcal {F}}} _X \right\} \quad \left( =\max \{ {{\,\mathrm{{rank}}\,}}({{{\mathcal {R}}} _{{{\mathcal {G}}} }}(X)) \,|\, X \in {{\mathcal {F}}} _X \}\right) \\ {} &{}=&{} \max \{ {{\,\mathrm{{rank}}\,}}(X) \,|\, X\in {{\mathcal {F}}} _X \cap A_{{{\mathcal {G}}} } \} \\ {} &{}=&{} \max \{ {{\,\mathrm{{rank}}\,}}({\tilde{\mathbf{{\mathcal {B}}} }}^*(x)) \,|\, {\tilde{\mathbf{{\mathcal {B}}} }}^*(x) \in {\mathcal {S}}_x\}. \end{array} \end{aligned}$$

#### Proof

Let $X \in {{\mathcal {F}}} _X$ be the matrix with maximum rank *r*. Then *X* is in the relative interior of the minimal face $f\unlhd {\mathbb {S}}_+^n$ containing ${\mathcal {F}}_{X}$, i.e.,

$$\begin{aligned} X\in {{\,\mathrm{{relint}}\,}}(f) = \begin{bmatrix} V&U \end{bmatrix} \begin{bmatrix} {\mathbb {S}}^r_{++} &{}\quad 0\\ 0 &{}\quad 0 \end{bmatrix} \begin{bmatrix} V&U \end{bmatrix}^{{{\,\mathrm{{\mathrm {T}}}\,}}}, \, \text { for some orthogonal } \begin{bmatrix} V&U \end{bmatrix}. \end{aligned}$$

The nonsingular congruence $P^{T}XP$ is feasible for each $P \in {{\mathcal {G}}}$, and also has rank *r*. Note that

$$\begin{aligned} A,B \in {\mathbb {S}}_+^n\implies {{\,\mathrm{{rank}}\,}}(A+B) \ge \max \{ {{\,\mathrm{{rank}}\,}}(A), {{\,\mathrm{{rank}}\,}}(B)\}. \end{aligned}$$

Therefore, applying the Reynolds operator, we have

$$\begin{aligned} X_{0} = {\frac{1}{|{{\mathcal {G}}} |}\sum _{P \in {{\mathcal {G}}} }P^{T}XP} \in {{\,\mathrm{{relint}}\,}}(f). \end{aligned}$$

Since $X_{0} \in {{{\mathcal {A}}}} _{{{\mathcal {G}}} }$, we have $Q^{T}X_{0}Q \in {\mathcal {S}}_x\,(= Q^T({{\mathcal {F}}} _X \cap A_{{{\mathcal {G}}} })Q)$ and it has rank *r*, where *Q* is the orthogonal matrix given above in (2.10).

Conversely, if ${\tilde{\mathbf{{\mathcal {B}}} }}^*(x) \in {\mathcal {S}}_x$ with rank *r*, then $X := Q {\tilde{\mathbf{{\mathcal {B}}} }}^*(x) Q^{T}$ is in ${\mathcal {F}}_{X}$ with rank *r*. $\square$

Note that in the proof of Theorem 3.1 we exploit the following known properties of the Reynolds operator: ${{\,\mathrm{{rank}}\,}}({{{\mathcal {R}}} _{{{\mathcal {G}}} }}(X)) \ge {{\,\mathrm{{rank}}\,}}(X),$ which is valid for all *X* that are positive semidefinite, and ${{{\mathcal {R}}} _{{{\mathcal {G}}} }}(F_X) ={\mathcal {F}}_X\cap A_{{\mathcal {G}}}$.

#### Corollary 3.2

The program (2.1) is strictly feasible if, and only if, its symmetry reduced program (2.14) is strictly feasible.

#### Remark 3.3

From the proof of Theorem 3.1, if there is a linear transformation $X = {{\mathcal {L}}} (x)$ with a full rank feasible ${\hat{X}} \in {{\,\mathrm{range}\,}}({{\mathcal {L}}} ), {\hat{X}} = {{\mathcal {L}}} ({\hat{x}})$, then in general we can conclude that the substitution $X={{\mathcal {L}}} (x)$ results in a *smaller* **SDP** with strict feasibility holding at ${\hat{x}}$, i.e.,

$$\begin{aligned} {\hat{X}}\succ 0, {{\mathcal {A}}}({\hat{X}})=b, {\hat{X}} = {{\mathcal {L}}} ({\hat{x}}) \implies {{\mathcal {L}}} ({\hat{x}})\succ 0, ({{\mathcal {A}}}\circ {{\mathcal {L}}} )({\hat{x}}) = b. \end{aligned}$$

### Exposing vectors

For many given combinatorial problems, the semidefinite relaxation is not strictly feasible, i.e., it is degenerate, ill-posed, and we can apply **FR** [18, 46, 55]. From Section 3.1 above, we see that this implies that the symmetry reduced problem is degenerate as well. Although both **SR** and **FR** can be performed separately to obtain two independent problems, there has not been any study that implements these techniques simultaneously and efficiently for **SDPs** , i.e., to obtain a symmetry reduced problem that also guarantees strict feasibility. Recall that [39] combines partial **FR** and **SR** for solving **SOS** programs.

In what follows, we show that the exposing vectors of the symmetry reduced program (2.14) can be obtained from the exposing vectors of the original program (2.1). This enables us to facially reduce the symmetry reduced program (2.14) using the structure from the original problem.

Let $W = UU^{T}$, with $U\in {\mathbb {R}}^{n\times (n-r)}$ full column rank; and let *W* be a nonzero exposing vector of a face of ${\mathbb {S}}_+^n$ containing the feasible region ${{\mathcal {F}}} _X$ of (2.1).^4^ Let $V \in {\mathbb {R}}^{n \times r}$ be such that

$$\begin{aligned} {{\,\mathrm{range}\,}}(V) = {{\,\mathrm{null}\,}}(U^T). \end{aligned}$$

Then **FR** means that we can use the substitution $X={{\mathcal {V}}} ^*(R)=VRV^T$ and obtain the following equivalent, smaller, formulation of (2.1):

3.1 $$\begin{aligned} p^*_{{\textbf {SDP}}\,} = \min \{ \langle V^{T}CV,R \rangle \;|\; \langle V^{T}A_{i}V,R \rangle = b_{i}, \;\; i \in {{\mathcal {I}}}\subseteq \{1,\ldots ,m\}, \;\; R \in {\mathbb {S}}^r_+\}.\nonumber \\ \end{aligned}$$

If *W* exposes the minimal face containing ${{\mathcal {F}}} _X$, then strict feasibility holds. In fact, ${\hat{R}}$ strictly feasible corresponds to ${\hat{X}}={{\mathcal {V}}} ^*({\hat{R}}) \in {{\,\mathrm{{relint}}\,}}({{\mathcal {F}}} _X)$.

The following results show how to find an exposing vector that is in the ${\text {commutant}\,A_{{{\mathcal {G}}} }}$.

#### Lemma 3.4

Let *W* be an exposing vector of rank *d* of a face ${{\mathcal {F}}} \unlhd {\mathbb {S}}_+^n, {{\mathcal {F}}} _X\subseteq {{\mathcal {F}}}$. Then there exists an exposing vector $W_{{{\mathcal {G}}} } \in A_{{{\mathcal {G}}} }$ of ${{\mathcal {F}}}$ with ${{\,\mathrm{{rank}}\,}}(W_{{{\mathcal {G}}} }) \ge d$.

#### Proof

Let *W* be the exposing vector of rank *d*, i.e.,

3.2 $$\begin{aligned} {W\succeq 0 \text { and }} \left( X \in {{\mathcal {F}}} \implies \langle W , X \rangle = 0\right) . \end{aligned}$$

Since (2.1) is ${{\mathcal {G}}}$-invariant, $PXP^{T} \in {{\mathcal {F}}} _X$ for every $P \in {{\mathcal {G}}}$, we conclude that

$$\begin{aligned} \langle W, PXP^{T} \rangle = \langle P^{T}WP, X \rangle = 0. \end{aligned}$$

Therefore, $P^{T}WP \succeq 0$ is an exposing vector of rank *d*. Thus $W_{{{\mathcal {G}}} } = \frac{1}{|{{\mathcal {G}}} |} \sum _{P \in {{\mathcal {G}}} } P^{T}WP$ is an exposing vector of ${{\mathcal {F}}}$.

That the rank is at least *d* follows from taking the sum of nonsingular congruences of $W\succeq ~0$. $\square$

Lemma 3.4 shows that $A_{{{\mathcal {G}}} }$ contains exposing vectors. This result is a valuable addition to the list of objects that exhibit symmetry, see for example: dual solutions and the central path in [31]; solutions on the central path and some search directions of primal-dual interior-point methods, in [29]; and infeasibility certificates, in [45].

Note that one can obtain an exposing vector $W_{{{\mathcal {G}}} } \in A_{{{\mathcal {G}}} }$ from an exposing vector *W* by using the Reynolds operator, as done in Lemma 3.4. However, in some cases $W_{{{\mathcal {G}}} }$ can be more easily derived, as our examples in the later numerical sections show. We now continue and show that $Q^{T}W_{{{\mathcal {G}}} } Q$ is also an exposing vector.

#### Lemma 3.5

Let $W \in A_{{{\mathcal {G}}} }$ be an exposing vector of a face ${{\mathcal {F}}} \unlhd {\mathbb {S}}_+^n, {{\mathcal {F}}} _X\subseteq {{\mathcal {F}}}$, Let *Q* be the orthogonal matrix given above in (2.10). Then ${\widetilde{W}} = Q^{T}WQ$ exposes a face of ${\mathbb {S}}_+^n$ containing ${\mathcal {S}}_x$.

#### Proof

Let

$$\begin{aligned} Z =\sum _{i=1}^{d} x_i {{\tilde{B}}}_i = Q^{T}\left( \sum _{i=1}^{d} x_i B_i\right) Q \in {\mathcal {S}}_x. \end{aligned}$$

Then, by construction *Z* is a block-diagonal matrix, say $Z = {{\,\mathrm{{Blkdiag}}\,}}(Z_{1},\ldots ,Z_{t})$. Now, since *W* is an exposing vector of the face of ${\mathbb {S}}_+^n$ containing ${{\mathcal {F}}} _X$ we have

$$\begin{aligned} \begin{array}{rcll} WX = 0, ~\forall X \in {{\mathcal {F}}} _X &{}\implies &{} WX = 0, &{} \forall X =\sum \limits _i x_i B_i\succeq 0, \text{ for } \text{ some } x \text { with } Ax=b \\ &{}\implies &{} {\widetilde{W}}Z = 0, &{} \forall Z \in {\mathcal {S}}_x, \\ \end{array} \end{aligned}$$

where ${\widetilde{W}} = Q^{T}WQ \succeq 0$. Thus, ${\widetilde{W}}$ is an exposing vector of a proper face of ${\mathbb {S}}_+^n$ containing ${\mathcal {S}}_x$.

Since $Z = {{\,\mathrm{{Blkdiag}}\,}}(Z_{1},\ldots ,Z_{t})$ is a block-diagonal matrix and $W \in A_{{{\mathcal {G}}} }$, we have that ${\widetilde{W}} = {{\,\mathrm{{Blkdiag}}\,}}({\widetilde{W}}_{1},\ldots ,{\widetilde{W}}_{t})$ with ${\widetilde{W}}_{i}$ the corresponding *i*-th diagonal block of $Q^{T}WQ$. $\square$

Since we may assume $W \in A_{{{\mathcal {G}}} }$, the exposing vector $Q^{T}WQ$ is a block-diagonal matrix. Now, let us show that $Q^{T}WQ$ exposes the minimal face of ${\mathbb {S}}_+^n$ containing ${\mathcal {S}}_x$, ${{\,\mathrm{face}\,}}({\mathcal {S}}_x)$. It suffices to show that the rank of $Q^{T}WQ$ is $n-r$, see Theorem 3.1.

#### Theorem 3.6

Let $W\in A_{{{\mathcal {G}}} }$ be an exposing vector of ${{\,\mathrm{face}\,}}({{\mathcal {F}}} _X)$, the minimal face of ${\mathbb {S}}_+^n$ containing ${{\mathcal {F}}} _X$. Then the block-diagonal matrix ${\widetilde{W}} = Q^{T}WQ$ exposes ${{\,\mathrm{face}\,}}({\mathcal {S}}_x)$, the minimal face of ${\mathbb {S}}_+^n$ containing ${\mathcal {S}}_x$.

#### Proof

The minimality follows from Theorem 3.1, as ${{\,\mathrm{{rank}}\,}}({\widetilde{W}})={{\,\mathrm{{rank}}\,}}(W) = n-r$. $\square$

Now let ${\widetilde{W}} = Q^{T}WQ$ expose the minimal face of ${\mathbb {S}}_+^n$ containing ${\mathcal {S}}_x$, and let

$$\begin{aligned} {\widetilde{W}} ={{\,\mathrm{{Blkdiag}}\,}}({\widetilde{W}}_{1},\ldots ,{\widetilde{W}}_{t}), \quad {\widetilde{W}}_{i}={\tilde{U}}_i{\tilde{U}}_i^T, \,{{\tilde{U}}}_i \text { full rank, } \,i=1,\ldots ,t. \end{aligned}$$

Let ${\tilde{V}}_i$ be a full rank matrix whose columns form a basis for the orthogonal complement to the columns of ${\tilde{U}}_{i}, i=1,\ldots ,t$. Take ${\tilde{V}} = {{\,\mathrm{{Blkdiag}}\,}}({\tilde{V}}_{1},\ldots ,{\tilde{V}}_{t})$. Then, the facially reduced formulation of (2.14) is

3.3 $$\begin{aligned} \begin{aligned} p^*_{FR} =&\min \{ c^{T}x \;|\; {Ax=b}, \;\; {\tilde{\mathbf{{\mathcal {B}}} }}^*(x) = {\tilde{V}}{\tilde{R}}{\tilde{V}}^{T}, \; {\tilde{R}} \succeq 0\}\\ =&\min \{ c^{T}x \;|\; {Ax=b}, \;\; {\tilde{\mathbf{{\mathcal {B}}} }}^*_{k}(x) = {\tilde{V}}_{k}{\tilde{R}}_{k}{\tilde{V}}_{k}^{T}, \; {\tilde{R}}_{k} \succeq 0, \; \forall k = 1,\ldots ,t\}, \end{aligned} \end{aligned}$$

where ${\tilde{V}}_{k}{\tilde{R}}_{k}{\tilde{V}}_{k}^{T}$ is the corresponding *k*-th block of ${\tilde{\mathbf{{\mathcal {B}}} }}^*(x)$, and ${\tilde{R}} = {{\,\mathrm{{Blkdiag}}\,}}({\tilde{R}}_{1},\ldots ,{\tilde{R}}_{t})$. Note that some of the blocks ${\tilde{\mathbf{{\mathcal {B}}} }}^*_{k}(x)$, and corresponding ${\tilde{R}}_{k}$, might be the same and thus can be removed in the computation, see Theorem 2.6.

#### Remark 3.7

We have assumed that an exposing vector of the minimal face of the original **SDP** (2.1) is available. If this is not the case, then we can find a strictly feasible formulation of (2.1), and an exposing vector of the minimal face for the original problem, by using a finite number (at most $\min \{m,n-1\}$) facial reduction steps, e.g., [18, 55, 56].

We note here that reduction techniques based on the Constrained Set Invariance Conditions, such as $*$-algebra techniques, can obtain strict feasibility by removing zero blocks after the appropriate projection, see [46].

#### Order of reductions

To obtain the combined symmetry and facially reduced semidefinite program (3.3), we first apply **SR** to (2.1), and then follow this with **FR** to the form in (2.14). A natural question is whether the order of reduction matters.

Note that the objective $\langle V^{T}CV, R \rangle$ and the constraints $\langle V^{T}A_{i}V,R \rangle = b_{i}, \; i \in {{\mathcal {I}}}\subseteq \{1,\ldots ,m\}$, of the facially reduced program (2.3), see also (3.1), depend on the choice of *V*. We now show that the choice of this *V* is crucial when reversing the order of reductions **FR** and **SR**. For a naive choice of *V*, we can lose all symmetries structure for **SR** in Sect. 2.2. For example, assume the data matrices $C,A_{1},\ldots ,A_{m}$ of the original problem are invariant under a non-trivial permutation group ${{\mathcal {G}}}$, i.e., they are in the commutant ${{\mathcal {A}}}_{{\mathcal {G}}}$, see (2.4). However the data matrices $V^{T}CV,V^{T}A_{1}V,\ldots ,V^{T}A_{m}V$ of the facially reduced problem may not be invariant under any non-trivial group of permutation matrices for the given *V*. Note that we can always replace $V \leftarrow VS$ using any invertible *S*. Then an arbitrary invertible congruence $S^TV^TA_iVS$ will destroy the symmetry structure in the constraint matrices.

##### Lemma 3.8

Let $V, {\tilde{V}}, Q$ be given as in the above paragraph, and in Theorem 3.6 and (3.3). Then

$$\begin{aligned} {{\,\mathrm{range}\,}}(V) = {{\,\mathrm{range}\,}}(Q{\tilde{V}}). \end{aligned}$$

##### Proof

$$\begin{aligned} \begin{array}{rcl} {{\,\mathrm{range}\,}}({\tilde{V}}) = {{\,\mathrm{null}\,}}(Q^{T}WQ) &{}\implies &{} Q({{\,\mathrm{range}\,}}({\tilde{V}})) = Q({{\,\mathrm{null}\,}}(Q^{T}WQ)) \\ {} &{}\implies &{} {{\,\mathrm{range}\,}}(Q{\tilde{V}}) = {{\,\mathrm{null}\,}}(Q^{T}WQQ^T) \\ {} &{}\implies &{} {{\,\mathrm{range}\,}}(Q{\tilde{V}}) = {{\,\mathrm{null}\,}}(W). \end{array} \end{aligned}$$

$$\square $$

From Lemma 3.8, we can set $V = Q{\tilde{V}}$ for **FR**. The objective and constraints become

$$\begin{aligned} \langle V^{T}CV ,R \rangle = \langle {\tilde{V}}^{T}{\tilde{C}}{\tilde{V}} ,R \rangle , \quad \langle V^{T}A_{i}V ,R \rangle = \langle {\tilde{V}}^{T}{\tilde{A}}_{i}{\tilde{V}},R \rangle = b_i, \forall i. \end{aligned}$$

As ${\tilde{C}},{\tilde{A}}_{i}$ and ${\tilde{V}}$ are block-diagonal matrices with appropriate sizes, the data matrices ${\tilde{V}}^{T}{\tilde{C}}{\tilde{V}}$ and ${\tilde{V}}^{T}{\tilde{A}}_{i}{\tilde{V}}$ are block-diagonal as well. Since *R* is also a block-diagonal matrix, this choice of *V* implicitly exploits symmetry of the original problem data. The reduction in this case is a special case of a general reduction technique known as a projection-based method, see [46] and Remark 2.1 above.

We conclude that if **FR** is implemented first, then for **SR** to follow it is crucial to find a suitable matrix *V* to retain the symmetry structure in the facially reduced problem. Therefore, it is more convenient for our approach to apply symmetry reduction before facial reduction and exploit the simple relation with the exposing vectors. However, for some other symmetry reduction methods it might be more appropriate to do first **FR** and then **SR** , assuming that some symmetry is preserved after **FR** and that the **SR** and **FR** procedures have comparable costs, see e.g., [39].

### Doubly nonnegative, **DNN**, program

In this section, the theory above for **SDP** is extended to doubly nonnegative program. We show that if a maximal exposing vector *W* (see Definition 3.11) of the **DNN** program (3.4) is given, then we can construct an exposing vector for the minimal face of the symmetry reduced **DNN** program (3.5). This results in a strictly feasible symmetry reduced **DNN** program (3.6).

Note that in addition to positive definiteness, we need $X > 0$, elementwise positivity, for strict feasibility to hold for the **DNN** relaxation. We denote the cone of nonnegative symmetric matrices of order *n* by ${{{\mathbb {N}}}}^n$. The following extends [27, Proposition 2.3] for the intersection of faces to include exposing vectors.

#### Theorem 3.9

Let $F_S \unlhd {\mathbb {S}}_+^n$, and let $F_N \unlhd {{{\mathbb {N}}}}^n$. Let $W_S\in {\mathbb {S}}_+^n, W_N\in {{{\mathbb {N}}}}^n$ be exposing vectors for $F_S,F_N$, respectively. Then

$$\begin{aligned} W_S+W_N \text { is an exposing vector for } F_S\cap F_N \text { for } {\textbf {DNN}}\,^n. \end{aligned}$$

#### Proof

Note that since ${{{\mathbb {N}}}}^n$ is a polyhedral cone, and both ${\mathbb {S}}_+^n,{{{\mathbb {N}}}}^n$ are self-dual, we get that the *dual cone* (nonnegative polar)

$$\begin{aligned} ({\textbf {DNN}}\,^n)^* = {\mathbb {S}}_+^n+ {{{\mathbb {N}}}}^n. \end{aligned}$$

Note that, by abuse of notation,

$$\begin{aligned} \langle W_S,F_S\rangle = 0, \quad \langle W_N,F_N\rangle = 0. \end{aligned}$$

We can now take the sum of the exposing vectors and it is clearly an exposing vector on the intersection of the faces. $\square$

#### Remark 3.10

   

1. Theorem 3.9 holds for arbitrary closed convex cones.
2. For our application, we note that the intersection $F_S\cap F_N$ is characterized by the facial representation $X\in V{\mathbb {S}}^r_+ V^T$ and $X_{ij}=0$ for appropriate indices $i,\!j$. **FR** on the **PSD** cone allows one to obtain a new **SDP** problem of lower dimension, since any face of the **PSD** cone is isomorphic to a smaller **PSD** cone. However, the **DNN** cone does not possess this property. Namely, a face of a **DNN** cone is not necessarily isomorphic to a smaller **DNN** cone. However, our splitting still allows for a simplification based on the Cartesian product of a **SDP** cone and a ${{{\mathbb {N}}}}^n$ cone, see (2.9), the paragraph after (2.9), and Remark 5.1, below.

The **DNN** program is defined as

3.4 $$\begin{aligned} ({{{\mathcal {P}}} _{\textbf {DNN}}\,}) \qquad p^*_{{\textbf {DNN}}\,} := \min \{ \langle C,X \rangle \;|\; {{{\mathcal {A}}}(X)}=b, \, X \in {\textbf {DNN}}\,^n\}. \end{aligned}$$

The symmetry reduced formulation of the **DNN** program (3.4) is

3.5 $$\begin{aligned} p^*_{{\textbf {DNN}}\,}= \min \{ c^{T}x \;|\; {Ax=b}, \, x \ge 0,\ {\tilde{\mathbf{{\mathcal {B}}} }}^*_{k}(x) \succeq 0, \, k = 1,\ldots ,t \}, \end{aligned}$$

see (2.11) for the definition of ${\tilde{\mathbf{{\mathcal {B}}} }}^*_{k}(x)$. Recall that the symmetry reduced formulation of the **SDP** program (2.1) is (2.14). The ambient cone of the symmetry reduced program (3.5) is the Cartesian product of cones $({\mathbb {R}}^{d}_{+},{\mathbb {S}}_+^{n_1},\ldots ,{\mathbb {S}}_+^{n_t})$.

Let $W \in {\textbf {DNN}}\,^{*}$ be an exposing vector of (3.4). Then $W = W_{S} + W_{N}$ for some $W_{S} \in {\mathbb {S}}_+^n$ and $W_{N} \in {{{\mathbb {N}}}}^n$. The exposing vector $W \in {\textbf {DNN}}\,^{*}$ satisfies $\langle W , X \rangle = 0$ for every feasible *X* of (3.4). Since it also holds that $\langle W_{S}, X\rangle \ge 0$ and $\langle W_{N} , X \rangle \ge 0$, we have

$$\begin{aligned} \langle W_{S}, X\rangle = \langle W_{N} , X \rangle = 0, \end{aligned}$$

for every feasible *X* of (3.4).

We are going to construct an exposing vector for the symmetry reduced program (3.5) by using *W*. Here the exposing vectors $(\widetilde{W_{n_{1}}},\ldots ,\widetilde{W_{n_{t}}})$ for the semidefinite cones $({\mathbb {S}}^{n_1},\ldots ,{\mathbb {S}}^{n_t})$ can be derived in the same way as before. Therefore we only have to find an exposing vector for the nonnegative cone ${\mathbb {R}}^{d}_+$. Let *x* be feasible for (3.5). Then $X = \mathbf{{\mathcal {B}}} ^{*}(x)$ is feasible for (3.4). We have

$$\begin{aligned} \langle W_{N} , X \rangle = \langle W_{N} , \mathbf{{\mathcal {B}}} ^{*}(x) \rangle = \langle \mathbf{{\mathcal {B}}} (W_{N}), x \rangle = 0. \end{aligned}$$

Define $w := \mathbf{{\mathcal {B}}} (W_{N})$. Since $W_{N}$ is nonnegative and $\left( \mathbf{{\mathcal {B}}} (W_{N})\right) _{i} = \langle B_{i}, W_{N} \rangle$ for some zero-one matrix $B_{i}$, the vector *w* is nonnegative. Then $\langle w,x\rangle = 0$ implies that *w* is an exposing vector for the cone ${\mathbb {R}}^{d}_+$ of (3.5).

Thus facial reduction for the nonnegative cone ${\mathbb {R}}_{+}^{d}$ simply removes the entries $x_{i}$ associated to positive entries $w_{i} > 0$ from the program. Let $\bar{x}$ be the vector obtained by removing these entries from *x*. Define the new data matrices $\bar{c},\bar{A}$ correspondingly. The facial reduction for the semidefinite cones are handled in the same way as before. This yields the following facially reduced formulation of (3.5)

3.6 $$\begin{aligned} \begin{aligned} p^*_{{\textbf {DNN}}\,} =&\min \{ \bar{c}^{T}\bar{x} \;|\; {\bar{A}\bar{x}=b}, \, \bar{x} \ge 0, \, {\tilde{\mathbf{{\mathcal {B}}} }}^*_{k}(\bar{x}) = {\tilde{V}}_{k}{\tilde{R}}_{k}{\tilde{V}}_{k}^{T}, \, {\tilde{R}}_{k} \succeq 0, \, \forall k = 1,\ldots ,t\}. \end{aligned} \end{aligned}$$

In the sequel, we denote by ${{\,\mathrm{{supp}}\,}}(M)$ the support of the matrix *M*.

#### Definition 3.11

An exposing vector *W* of the ${\textbf {DNN}}\,$ program (3.4) is a maximal exposing vector if it has a decomposition $W = W_{S} + W_{N}$ for some $W_{S} \in {\mathbb {S}}_+^n$ and $W_{N} \in {{{\mathbb {N}}}}^n$ satisfying:

(i) $${{\,\mathrm{{rank}}\,}}( W_{S})$$ is maximal;
(ii) the number of positive entries in $W_{N}$ (support) is maximal.

Note that if $W = W_S+W_N, W^\prime = W_S^\prime +W_N^\prime$, are maximal exposing vectors of a **DNN** program, with corresponding appropriate decompositions, then $W_S, W_S^\prime , W_N, W_N^\prime$ are also exposing vectors of faces containing the minimal face. Moreover, $W + W^\prime = W_S + W_S^\prime + W_N + W_N^\prime$ is also a maximal exposing vector. Therefore the rank of $W_S$ and the support of $W_N$, in the decomposition of a maximal exposing vector *W* in Definition 3.11, are in addition both *unique*.

#### Theorem 3.12

Assume that *W* is a maximal exposing vector of the ${\textbf {DNN}}\,$ relaxation (3.4) with decomposition $W=W_S+W_N$, and $W_S$ exposes the minimal face of the **SDP** relaxation (2.1). Then we can find the minimal face of the symmetry reduced program (3.5). Or equivalently, the facially and symmetry reduced program (3.6) is strictly feasible.

#### Proof

Assume, for the sake of contradiction, that (3.6) is not strictly feasible. The existence of a feasible solution for (3.6) such that ${\tilde{R}}_{k} \succ 0$ for $k = 1,\ldots ,t$ can be derived using ${{\tilde{V}}}$ in the same way as before, see Theorem 3.6. Țhe main point is that as for Theorem 3.6, we use the exposing vector to get a face $F_S$ that might not be a proper face of the minimal face of the **SDP** relaxation. Therefore, we consider here only the case that there does not exist feasible $\bar{x}$ for (3.6) that is strictly positive. Then there exists an exposing vector $w^\prime \in {\mathbb {R}}_{+}^{d}$ for (3.5) such that ${{\,\mathrm{{supp}}\,}}(w) \subsetneq {{\,\mathrm{{supp}}\,}}(w^\prime )$. Let $W_{N}^\prime := \mathbf{{\mathcal {B}}} ^{*}(w^\prime ) \in {{{\mathbb {N}}}}^n$. Then ${{\,\mathrm{{supp}}\,}}(W_{N}) \subsetneq {{\,\mathrm{{supp}}\,}}(W_{N}^\prime )$. Let $X \in {\textbf {DNN}}\,$ be feasible for (3.4). Then ${{{\mathcal {R}}} _{{{\mathcal {G}}} }}(X) = \mathbf{{\mathcal {B}}} ^{*}(x) \in {\textbf {DNN}}\,$ for some *x* feasible for (3.5), and thus

$$\begin{aligned} \langle W_{N}^\prime ,{{{\mathcal {R}}} _{{{\mathcal {G}}} }}(X) \rangle = \langle w^\prime ,x \rangle = 0. \end{aligned}$$

But ${{\,\mathrm{{supp}}\,}}(X) \subseteq {{\,\mathrm{{supp}}\,}}({{{\mathcal {R}}} _{{{\mathcal {G}}} }}(X))$, this means that $\langle W_{N}^\prime , X \rangle = 0$. Thus $W_{N}^\prime$ is an exposing vector for (3.4) such that ${{\,\mathrm{{supp}}\,}}(W_{N}) \subsetneq {{\,\mathrm{{supp}}\,}}(W_{N}^\prime )$. This contradicts the maximality of *W*. Thus the program (3.6) is strictly feasible. Note that the fact that we could move the nonnegativity to the reduced variable *x* was essential for obtaining the Slater condition. $\square$

#### Remark 3.13

One can also prove Theorem 3.12 by exploiting the following properties of ${{{\mathcal {R}}} _{{{\mathcal {G}}} }}(X)$, the Reynolds operator of ${{{\mathcal {G}}} }$, see (2.5). For all feasible *X*, it holds that

$$\begin{aligned} {{\,\mathrm{{rank}}\,}}\left( {{{\mathcal {R}}} _{{{\mathcal {G}}} }}(X)\right) \ge {{\,\mathrm{{rank}}\,}}(X),~ {{\,\mathrm{{supp}}\,}}({{{\mathcal {R}}} _{{{\mathcal {G}}} }}(X)) \supseteq {{\,\mathrm{{supp}}\,}}(X). \end{aligned}$$

#### Facial reduction from the ground set for **DNN**

Our applications involve quadratic models of hard combinatorial problems. We now see that the view of strict feasibility and **FR** in [61, Theorems 3.1, 3.2] can be easily extended from **SDP** to **DNN**.

We follow the notation in [61] and define the feasible set or *ground set* of a quadratically constrained program as:

$$\begin{aligned} {{\mathcal {Q}}\,}:= \left\{ x \in {\mathbb {R}}^{n} \;|\; {\mathcal {A}}\left( \begin{bmatrix} 1 &{}\quad x^{T} \\ x &{}\quad xx^{T} \end{bmatrix}\right) = 0, \, x\ge 0 \right\} , \end{aligned}$$

where ${{\mathcal {A}}}$ is a linear transformation. The relaxation, lifting, is then given by

$$\begin{aligned} {\hat{{{\mathcal {Q}}\,}}} := \left\{ \begin{bmatrix} 1 &{}\quad x^{T} \\ x &{}\quad X \end{bmatrix} \in {\textbf {DNN}}\,^{n+1} \;|\; {\mathcal {A}}\left( \begin{bmatrix} 1 &{}\quad x^{T} \\ x &{}\quad X \end{bmatrix}\right) = 0 \right\} . \end{aligned}$$

Let the *gangster set* for ${{\mathcal {Q}}\,}$ be defined as

$$\begin{aligned} {{\mathcal {G}}} _{{\mathcal {Q}}\,}= \left\{ (i,j) : x_ix_j = 0, \, \forall x \in {{\mathcal {Q}}\,}\right\} \end{aligned}$$

with complement ${{\mathcal {G}}} _{{\mathcal {Q}}\,}^c$. Let the gangster set for ${{\hat{{{\mathcal {Q}}\,}}}}$ be defined as

$$\begin{aligned} {{\mathcal {G}}} _{{{\hat{{{\mathcal {Q}}\,}}}}} = \left\{ (i,j) : X_{ij}= 0, \, \text{ for } \text{ all } \, \begin{bmatrix} 1 &{}\quad x^{T} \\ x &{}\quad X \end{bmatrix} \in {{{\hat{{{\mathcal {Q}}\,}}}} } \right\} . \end{aligned}$$

Note that here the gangster sets are equal ${{\mathcal {G}}} _{{{\mathcal {Q}}\,}} ={{\mathcal {G}}} _{\hat{{\mathcal {Q}}\,}}$, with appropriate indices. However, for a general **DNN**, we are not given the ground set and the gangster set is defined for the lifted problem only.

In what follows we use $e_k$ *or* *e* when the meaning is clear, to denote the vector of all ones of order *k*.

##### Theorem 3.14

(Slater) Suppose that ${{\,\mathrm{{conv}}\,}}({{\mathcal {Q}}\,})$ is full dimensional and that ${{\mathcal {G}}} _{{\mathcal {Q}}\,}= \emptyset$. Then the Slater condition holds for ${\hat{{{\mathcal {Q}}\,}}}$.

##### Proof

By the assumption, we can choose the finite set of vectors

3.7 $$\begin{aligned} \left\{ v^{ij} \in {{\mathcal {Q}}\,}\,|\, v^{i,j}_iv^{i,j}_j > 0, \, \text { for each } (i,j)\in \{1,\ldots ,n\} \times \{1,\ldots ,n\} \right\} . \end{aligned}$$

As in [61], we choose an affine linear independent set $\{x_i\}_{i=1}^{n+1} \subseteq {{\mathcal {Q}}\,}$, and form the matrix by adding ones and the $v^{i,j}$ defined in (3.7):

$$\begin{aligned} V := \begin{bmatrix} {e_{n+1}^T} &{}\quad {e_{n^2}^T} \\ \begin{bmatrix} x^1, \ldots , x^{n+1} \end{bmatrix}&\quad \begin{bmatrix} v^{1,1}, v^{1,2}, \ldots , v^{n,n} \end{bmatrix} \end{bmatrix} \end{aligned}$$

We lift and get the Slater point $W:= VV^T\in {{\hat{{{\mathcal {Q}}\,}}}}, W\succ 0, W>0$. $\square$

We now extend this to obtain **FR**. We use our exposing vector viewpoint rather than the internal view in [61, Theorem 3.2]. We note that we can not move here the nonnegativity constraints onto *R* as is done for our applications after **SR**. Moreover, though the Slater condition holds for the **FR** feasible set in (3.8), it is not necessarily true that the *Mangasarian-Fromovitz constraint qualification* holds, since some of the linear equality constraints typically become redundant after **FR**. We can however discard redundant equality constraints.

##### Theorem 3.15

(facial reduction) Suppose that the affine hull, ${{\,\mathrm{{aff}}\,}}({{\,\mathrm{{conv}}\,}}({{\mathcal {Q}}\,}))= {{\mathcal {L}}}$ and $\dim ({{\mathcal {L}}} )=d$. Then there exist *A* and *b* with *A* full row rank such that

$$\begin{aligned} {{\mathcal {L}}} = \{x\in {\mathbb {R}}^n\,|\, Ax=b\}. \end{aligned}$$

Let $$U = \begin{bmatrix}-b^T \\ A^T\end{bmatrix}$$ and *V* be full column rank with ${{\,\mathrm{range}\,}}(V) = {{\,\mathrm{null}\,}}(U)$. Then there exists a Slater point ${\hat{R}}$ for the **FR**, **DNN** feasible set

3.8 $$\begin{aligned} {{\hat{{{\mathcal {Q}}\,}}}}_R = \left\{ R \in {\mathbb {S}}^{d+1} \,|\, R\succeq 0, \,(VRV^T)_{{{\mathcal {G}}} _{{\mathcal {Q}}\,}^c} \ge 0,\, (VRV^T)_{{{\mathcal {G}}} _{{\mathcal {Q}}\,}} = 0,\, {{\mathcal {A}}}\left( VRV^T\right) = 0 \right\} ,\nonumber \\ \end{aligned}$$

where $(VRV^T)_S$ is the vector with indices chosen from the set *S*, and

3.9 $$\begin{aligned} {\hat{R}}\succ 0, \,(V{\hat{R}}V^T)_{{{\mathcal {G}}} _{{\mathcal {Q}}\,}^c} > 0,\, (V\hat{R}V^T)_{{{\mathcal {G}}} _{{\mathcal {Q}}\,}} = 0,\, {{\mathcal {A}}}\left( V{\hat{R}}V^T\right) = 0. \end{aligned}$$

##### Proof

The proof is as for Theorem 3.14 after observing that $UU^T$ is an exposing vector. More precisely, from

3.10 $$\begin{aligned} Ax-b =0 \iff \begin{bmatrix} 1 \\ x \end{bmatrix}^T \begin{bmatrix} -b^T \\ A^T \end{bmatrix} =0 \iff \begin{bmatrix} 1 \\ x \end{bmatrix} \begin{bmatrix} 1 \\ x \end{bmatrix}^T \begin{bmatrix} -b^T \\ A^T \end{bmatrix} \begin{bmatrix} -b^T \\ A^T \end{bmatrix}^T =0,\nonumber \\ \end{aligned}$$

we see that $YUU^T=0$ for all lifted *Y*, and therefore also for the minimal face. Thus $UU^T$ is an exposing vector. The result follows after restricting the selection in (3.7) to the complement ${{\mathcal {G}}} _{{\mathcal {Q}}\,}^c$. $\square$

### Singularity degree

The *singularity degree* defined for the *semidefinite* feasibility problem ${{\mathcal {P}}} _F$ (2.2), and denoted by ${{\,\mathrm{sd}\,}}({{\mathcal {P}}} _F)$, is the minimum number of steps with a nonzero exposing vector, for the **FR** algorithm to terminate with the minimal face. For ${{\mathcal {P}}} _{\textbf {SDP}}\,$ this means we terminate with a strictly feasible problem. Singularity degree was introduced for ${{\mathcal {P}}} _{\textbf {SDP}}\,$ in [58] to show that **SDP** feasibility problems always admit a *Hölder error bound*,^5^ more precisely, let $d={{\,\mathrm{sd}\,}}({{\mathcal {P}}} _F)$, ${{\mathcal {L}}} = \{X \,|\, {{\mathcal {A}}}(X) = b \}$, $U\subset {\mathbb {S}}^n$ be compact. Let ${{\,\mathrm{dist}\,}}$ denote the norm-distance to a set. Then it is shown in [58] that there exists $c>0$ such that

$$\begin{aligned} {{\,\mathrm{dist}\,}}(X,{{\mathcal {F}}} _X) \le c\left( {{\,\mathrm{dist}\,}}^{2^{-d}}(X,{\mathbb {S}}_+^n)+ {{\,\mathrm{dist}\,}}^{2^{-d}}(X,{{\mathcal {L}}} ) \right) , \, \forall X\in U. \end{aligned}$$

Remarkably, the exponent $2^{-d}$ is independent of *m*, *n* and the rank of the matrices in ${{\mathcal {F}}} _X$. It strongly indicates the importance of **FR** for **SDP**, especially when obtaining approximate solutions with splitting type methods. This is illustrated by our numerical results, see Table 7 below, where lower bounds obtained by **ADMM** are dramatically better than those for **IPM**.

In this section, we show that the singularity degree of a symmetry reduced program is equal to the singularity degree of the original problem, see Theorem 3.17. Thus, we provide a heuristic indication that this error measure does not grow when applying **SR**. Of course, after completing **FR**, the singularity degree is optimal, 0.

The facial reduction algorithm applied to the semidefinite program (2.1) is described as follows. At the *k*-th step, **FR** finds an exposing vector of the feasible region of the reduced **SDP** of (2.1)

3.11 $$\begin{aligned} \{ R \in {\mathcal {S}}^{r_{k}}_{+} \;|\; {\mathcal {A}}_{V}(R) = b_{{\mathcal {I}}}\}, \text { with } {\mathcal {A}}_{V}(R) = \left( \langle V^{T}A_{i}V ,R \rangle \right) _{i\in {{\mathcal {I}}}} \in {\mathbb {R}}^{|{{\mathcal {I}}}|}, \, {{\mathcal {I}}}\subseteq \{1,\ldots ,m\}.\nonumber \\ \end{aligned}$$

Here *V* is a given matrix updated after each **FR** step. In the first step, *V* is the identity matrix and (3.11) is the feasible region ${{\mathcal {F}}} _X$ of the original problem (2.1). An exposing vector is then obtained by solving the following *auxiliary system* for *y*:

3.12 $$\begin{aligned} 0 \ne {\mathcal {A}}_{V}^{*}(y) \succeq 0 \text { and } b^{T}y \le 0. \end{aligned}$$

If *y* exists, then $W = A_{V}^{*}(y) \in {\mathbb {R}}^{r_{k} \times r_{k}}$ is an exposing vector of the feasible region (3.11). We then do as follows:

3.13 $$\begin{aligned} \begin{array}{rl} (i) &{} \hbox {compute} V^\prime \in {\mathbb {R}}^{r_{k} \times r_{k+1}} \hbox {, full rank,} {{\,\mathrm{range}\,}}(V^\prime ) = {{\,\mathrm{null}\,}}(W); \\ (ii) &{} \text {set} V\leftarrow VV^\prime \in {\mathbb {R}}^{n \times r_{k+1}}; \\ (iii) &{} \text {repeat from (3.12)} . \end{array} \end{aligned}$$

At the *k*-th step, we have computed a vector *y* and a matrix $V^\prime$ that determines the facially reduced formulation at the next step. Choosing exposing vectors with maximum possible rank leads to the fewest iterations, see e.g., [40, 56]. For completeness, we now show that the number of iterations in the facial reduction algorithm only depends on the choice of *y* and not on the choice of $V^\prime$.

#### Lemma 3.16

The total number of facial reduction steps does not depend on the choice of $V^\prime$ and *V* in (3.13).

#### Proof

Assume that *y* satisfies the auxiliary system (3.12) for the feasible region (3.11). If we replace $V \in {\mathbb {R}}^{n \times r_{k}}$ in (3.11), with $VS \in {\mathbb {R}}^{n \times r_{k}}$, for some invertible matrix $S \in {\mathbb {R}}^{r_{k} \times r_{k}}$, then the same vector *y* satisfies the new auxiliary system, as $b^{T}y \le 0$ and

$$\begin{aligned} W_{S} := {\mathcal {A}}_{VS}^{*}(y) = \sum _{i=1}^{m} (S^{T}V^{T}A_{i}VS)y_{i} = S^{T}{\mathcal {A}}_{V}^{*}(y)S = S^{T}WS \succeq 0. \end{aligned}$$

Since *S* is invertible, it holds that $W_{S} \ne 0$ and ${{\,\mathrm{{rank}}\,}}(W_{S}) = {{\,\mathrm{{rank}}\,}}(W)$. Thus, we obtain the same reduction in the problem size at the *k*-th step.

As ${{\,\mathrm{null}\,}}(W_{S}) = S^{-1}{{\,\mathrm{null}\,}}(W)$, we have $S^{-1}V^\prime \in {{\,\mathrm{null}\,}}(W_{S})$, where $V'$ satisfies ${{\,\mathrm{range}\,}}(V') = {{\,\mathrm{null}\,}}(W)$ as in (3.13). For any invertible matrix $T\in {\mathbb {R}}^{r_{(k+1)} \times r_{(k+1)}}$, we have that $V_{S}^\prime = S^{-1}V^\prime T \in {{\,\mathrm{null}\,}}(W_{S})$. Thus, in the second step of (3.13), we have

$$\begin{aligned} VS \leftarrow (VS)V_{S}^\prime = VSS^{-1}V^\prime T = (VV^\prime ) T. \end{aligned}$$

This means we can can repeat our argument to show the reduction at each subsequent step is the same. $\square$

Now we describe the facial reduction algorithm applied to the symmetry reduced program (2.14). The facial reduction algorithm at the *k*-th step considers the feasible region in variables $(x,{\tilde{R}}_{1},\ldots ,{\tilde{R}}_{t})$ determined by

3.14 $$\begin{aligned} \begin{array}{rcl} Ax &{}= &{}b\\ {{\,\mathrm{{blkdiag}}\,}}\left( {\tilde{\mathbf{{\mathcal {B}}} }}^*(x) \right) &{}= &{} \left( {\tilde{V}}_{1}{\tilde{R}}_{1}{\tilde{V}}_{1}^{T},\ldots ,{\tilde{V}}_{t}{\tilde{R}}_{t}{\tilde{V}}_{t}^{T}\right) \\ {\tilde{R}}_{k} &{}\in &{} {\mathcal {S}}_{+}^{{\tilde{r}}_{k}}, \end{array} \end{aligned}$$

for some ${\tilde{V}} = {{\,\mathrm{{Blkdiag}}\,}}({\tilde{V}}_{1},\ldots ,{\tilde{V}}_{t})$ with ${\tilde{V}}_{i} \in {\mathbb {R}}^{n_{i} \times {\tilde{r}}_{k}}$, see also (3.3). Here ${{\,\mathrm{{blkdiag}}\,}}= {{\,\mathrm{{Blkdiag}}\,}}^*$. In the first step, ${\tilde{V}}$ is the identity matrix and we obtain the feasible region ${{\mathcal {F}}} _{x}$ of the symmetry reduced program (2.14).

The auxiliary system for (3.14) is to find $(y,{\widetilde{W}}_{1},\ldots ,{\widetilde{W}}_{t})$ such that

3.15 $$\begin{aligned} \begin{array}{rll} A^{T}y &{}=&{} {\tilde{\mathbf{{\mathcal {B}}} }}({{\,\mathrm{{Blkdiag}}\,}}({\widetilde{W}}_{1},\ldots ,{\widetilde{W}}_{t}))\\ 0 &{}\ne &{} ({\tilde{V}}_{1}^{T}{\widetilde{W}}_{1}{\tilde{V}}_{1},\ldots , {\tilde{V}}_{t}^{T}{\widetilde{W}}_{t}{\tilde{V}}_{t} )\in ({\mathcal {S}}^{{\tilde{r}}_{1}}_+,\ldots ,{\mathcal {S}}^{{\tilde{r}}_{t}}_+) \text { and } b^{T}y \le 0. \end{array} \end{aligned}$$

Then ${{\,\mathrm{{Blkdiag}}\,}}({\tilde{V}}_{1}^{T}{\widetilde{W}}_{1}{\tilde{V}}_{1},\ldots , {\tilde{V}}_{t}^{T}{\widetilde{W}}_{t}{\tilde{V}}_{t} )$ is an exposing vector of the symmetry reduced problem. Let ${\tilde{V}}_{i}^\prime$ be the matrix whose independent columns span ${{\,\mathrm{null}\,}}({\tilde{V}}_{i}^{T}{\widetilde{W}}_{i}{\tilde{V}}_{i})$. In the facial reduction algorithm, we replace the matrix ${\tilde{V}}_{i}$ by ${\tilde{V}}_{i}{\tilde{V}}_{i}^\prime$. Then we repeat the algorithm until the auxiliary system (3.15) has no solution.

Our main result in this section is that the singularity degree of the symmetry reduced **SDP**  (2.14) is equal to the singularity degree of the original **SDP** (2.1).

#### Theorem 3.17

$${{\,\mathrm{sd}\,}}({{\mathcal {P}}} _{F_x}) = {{\,\mathrm{sd}\,}}({{\mathcal {P}}} _F).$$

#### Proof

We show first the inequality ${{\,\mathrm{sd}\,}}({{\mathcal {P}}} _{F_x}) \le {{\,\mathrm{sd}\,}}({{\mathcal {P}}} _F).$ In particular, we show that if we apply the facial reduction algorithm to the original SDP (2.1), then the solution of the auxiliary system (3.12) can be used to construct a solution to the auxiliary system (3.15) of the symmetry reduced problem (2.14).

Let *y* be a solution to the auxiliary system (3.12) in the *k*-th facial reduction step. Let $W = {{\mathcal {A}}}^{*}_V(y) \in A_{{{\mathcal {G}}} }$ (see Lemma 3.4) and ${\widetilde{W}} = Q^{T}WQ$, where *Q* is as specified in Theorem 2.6. Further, let ${\widetilde{W}}_{j} \in {\mathcal {S}}^{n_{j}}_+$ be the *j*-th block of *W* ($j=1,\ldots ,t$).

If $k=1$ in the **FR** algorithm, then the matrices *V* and ${\tilde{V}}$ are identity matrices. As $W \succeq 0$, we have ${\widetilde{W}}_{j} \succeq 0$ ($j=1,\ldots ,t$). It also holds that $b^{T}y \le 0$ and

3.16 $$\begin{aligned} {\tilde{\mathbf{{\mathcal {B}}} }}( {{\,\mathrm{{Blkdiag}}\,}}({\widetilde{W}}_{1},\ldots ,{\widetilde{W}}_{t})) = {\tilde{\mathbf{{\mathcal {B}}} }}(Q^{T}{{\mathcal {A}}}^{*}(y)Q) = \mathbf{{\mathcal {B}}} ({{\mathcal {A}}}^{*}(y)) = A^{T}y, \end{aligned}$$

see (2.10) and (2.13). Thus $(y,{\widetilde{W}}_{1},\ldots ,{\widetilde{W}}_{t})$ satisfies the auxiliary system (3.15). Also, we have that ${{\,\mathrm{{rank}}\,}}\left( {{\mathcal {A}}}^{*}(y)\right) = \sum _{j=1}^{t} {{\,\mathrm{{rank}}\,}}{\widetilde{W}}_{j}$. Let *V* and ${\tilde{V}} = {{\,\mathrm{{Blkdiag}}\,}}({\tilde{V}}_{1},\ldots ,{\tilde{V}}_{t})$ be matrices whose independent columns span ${{\,\mathrm{null}\,}}(W)$ and ${{\,\mathrm{null}\,}}({\widetilde{W}})$, respectively. It follows from Lemma 3.8 that ${{\,\mathrm{range}\,}}(V) = {{\,\mathrm{range}\,}}(Q{\tilde{V}})$. From Lemma 3.16 it follows that we can take $V= Q{\tilde{V}}$ in the next step.

Let $k > 1$ and $V= Q{\tilde{V}}$ where *V* and ${\tilde{V}}$ are derived in the previous iterate of the **FR** algorithm. Then, we have that

$$\begin{aligned} {{\mathcal {A}}}_{V}^{*}(y) = V^{T}{{\mathcal {A}}}^{*}(y)V = {\tilde{V}}^{T}\left( Q^{T}{{\mathcal {A}}}^{*}(y)Q\right) {\tilde{V}} = {\tilde{V}}^{T}{\widetilde{W}}{\tilde{V}} \end{aligned}$$

is block diagonal. As ${{\mathcal {A}}}_{V}^{*}(y) \succeq 0$, we have that each block ${\tilde{V}}_{j}^{T}{\widetilde{W}}_{j}{\tilde{V}}_{j}$ $(j=1,\ldots , t)$ is positive semidefinite. It also holds that $b^Ty\le 0$ and ${\tilde{\mathbf{{\mathcal {B}}} }}({\widetilde{W}}) = A^{T}y$. Thus $(y,{\widetilde{W}}_{1},\ldots ,{\widetilde{W}}_{t})$ satisfies the auxiliary system (3.15). Further, we have that ${{\,\mathrm{{rank}}\,}}\left( {{\mathcal {A}}}_{V}^{*}(y)\right) = \sum _{j=1}^{t} {{\,\mathrm{{rank}}\,}}({\tilde{V}}_{j}^{T}{\widetilde{W}}_{j}{\tilde{V}}_{j})$.

Let $V^\prime$ and ${\tilde{V}}_{j}^\prime$ $(j=1,\ldots , t)$ be matrices whose independent columns span ${{\,\mathrm{null}\,}}\left( {{\mathcal {A}}}_{V}^{*}(y)\right)$ and ${{\,\mathrm{null}\,}}({\tilde{V}}_{k}^{T}{\widetilde{W}}_{k}{\tilde{V}}_{k})$ $(j=1,\ldots , t)$, respectively. As ${{\mathcal {A}}}_{V}^{*}(y) = {\tilde{V}}^{T}{\widetilde{W}}{\tilde{V}}$ is block diagonal we can simply take $V^\prime = {\tilde{V}}^\prime$. Thus after updating $V \leftarrow VV^\prime$ and ${\tilde{V}} \leftarrow {\tilde{V}}{\tilde{V}}^\prime$, we have $V =Q {\tilde{V}}$ in the next step. We can repeat the same argument until the facial reduction algorithm terminates.

Next, we show that ${{\,\mathrm{sd}\,}}({{\mathcal {P}}} _{F_x}) \ge {{\,\mathrm{sd}\,}}({{\mathcal {P}}} _F)$. Let us assume that $(y,{\widetilde{W}}_{1},\ldots ,{\widetilde{W}}_{t})$ satisfies the auxiliary system (3.15) in the first facial reduction step. Recall that in the first step, ${\tilde{V}}$ is the identity matrix. For *Q* defined as in Theorem 2.6, we have that

3.17 $$\begin{aligned} W := Q{{\,\mathrm{{Blkdiag}}\,}}({\widetilde{W}}_{1},\ldots ,{\widetilde{W}}_{t})Q^{T} \in A_{{{\mathcal {G}}} }. \end{aligned}$$

To show that *y* satisfies the auxiliary system (3.12), we have to prove that ${{\mathcal {A}}}^{*}(y) \succeq 0$. It holds that

3.18 $$\begin{aligned} \mathbf{{\mathcal {B}}} ({{\mathcal {A}}}^{*}(y)) = A^{T}y = {\tilde{\mathbf{{\mathcal {B}}} }}({{\,\mathrm{{Blkdiag}}\,}}({\widetilde{W}}_{1},\ldots ,{\widetilde{W}}_{t})) = \mathbf{{\mathcal {B}}} (W), \end{aligned}$$

see also (3.16). The second equality above uses the feasibility of (3.15). Since we have that ${{\mathcal {A}}}^{*}(y) \in A_{{{\mathcal {G}}} }$ and $W \in A_{{{\mathcal {G}}} }$, it follows from (3.18) that $W = {{\mathcal {A}}}^{*}(y)$, and from (3.17) and (3.15) that ${{\mathcal {A}}}^{*}(y) \succeq 0$. Recall that we assumed that the data matrices of the **SDP** problem (2.1) are contained in the matrix $*$-algebra $A_{{{\mathcal {G}}} }$, see Sect. 2.2.

Let $k > 1$. Using $V = Q{\tilde{V}}$ where *V* and ${\tilde{V}}$ are derived in the previous iterate of the **FR** algorithm, (3.15) AND (3.17), we have that

$$\begin{aligned} 0 \preceq {\tilde{V}}^{T} {{\,\mathrm{{Blkdiag}}\,}}({\widetilde{W}}_{1},\ldots ,{\widetilde{W}}_{t}) {\tilde{V}} = V^{T}WV = V^{T}{{\mathcal {A}}}^{*}(y)V = {{\mathcal {A}}}_{V}^{*}(y). \end{aligned}$$

In addition, it follows from construction of *W* and Lemma 3.16 that we can take *V* and ${\tilde{V}}$ such that $V=Q{\tilde{V}}$ in the next **FR** step. $\square$

The following Corollary 3.18 follows from [61, Theorem 3.2] in that the linear manifold is represented by a concrete constraint and is applied to finding an exposing vector. More precisely, if we can find the affine span of our original feasible set in the ground space, then we can always find the representation using a linear mapping as in (3.10). This means we can always find the appropriate exposing vector and obtain singularity degree one, see also [18]. Note that this includes the hard combinatorial problems we work with below.

#### Corollary 3.18

Consider the quadratic model as given in Theorem 3.15, and suppose that the matrix *A* is part of the given data of the problem, Then the singularity degree is one.

#### Proof

The proof uses *A* to construct the exposing vector. Therefore, one step of the **FR** algorithm is sufficient, see (3.10). More precisely, the linear constraint in the ground set is lifted into the **SDP** as in (3.10). $\square$

#### Remark 3.19

The definition of singularity degree can be extended to **DNN**, and to a general cone optimization problem, to be the minimum number of steps in the **FR** [7, Algor. B]. Here this means we continue to find the minimum number of steps with nonzero exposing vectors. An interesting question is to find the relationship between the singularity degree of the **SDP** and the **DNN**. It appears that the **DNN** is at most one more than for **SDP**. Moreover it is known that the singularity degree of the ${\textbf {DNN}}\,^n$ is at most *n*, see [40, Corollary 20].

### Simplifications

After **FR** , some of the constraints become redundant in the facially reduced program (3.1). We show here that the same constraints are also redundant in the facially and symmetry reduced program (3.3). Proof of Lemma 3.20 is clear.

#### Lemma 3.20

For any subset ${\mathcal {I}} \subseteq [m] : = \{1,\ldots ,m\}$, we define the spectrahedron

$$\begin{aligned} {\mathcal {F}}({\mathcal {I}}):=\{ X \in {\mathcal {S}}^{n} \;|\; \langle A_i,X \rangle =b_i ~~\forall i\in {\mathcal {I}}, \; X = VRV^{T}, \; R \in {\mathcal {S}}^{r}_{+} \}. \end{aligned}$$

If the constraints in $[m]\backslash {\mathcal {I}}$ are redundant, e.g., ${\mathcal {F}}([m]) = {\mathcal {F}}({\mathcal {I}})$, then ${\mathcal {F}}([m]) \cap A_{{{\mathcal {G}}} } = {\mathcal {F}}({\mathcal {I}}) \cap A_{{{\mathcal {G}}} }$.

Although a proof of Corollary 3.21 follows directly from Lemma 3.20, we provide it due to our intricate notation.

#### Corollary 3.21

Let ${\mathcal {I}} \subsetneq \{1,\ldots ,m\}$. Suppose that the constraints $\langle A_{k},VRV^{T} \rangle = b_{k}, k \notin {\mathcal {I}}$, are redundant in (3.1), i.e., the facially reduced formulation (3.1) is equivalent to

3.19 $$\begin{aligned} \min _{R \in {\mathbb {S}}^r_+} \{ \langle V^{T}CV,R \rangle \;|\; \langle V^{T}A_{i}V,R \rangle = b_{i}, \;\; \forall i \in {\mathcal {I}} \}. \end{aligned}$$

Then the constraints

$$\begin{aligned} \sum _{j=1}^{d} A_{k,j}x_{j} = b_{k}, k \notin {\mathcal {I}}, \end{aligned}$$

are redundant in (3.3), i.e., the facially and symmetry reduced program (3.3) is equivalent to

3.20 $$\begin{aligned} \min _{x \in {\mathbb {R}}^{d}, {\tilde{R}} \in {\mathbb {S}}^r_+ } \left\{ c^{T}x \;|\; \sum _{j=1}^{d} A_{i,j}x_{j} = b_{i}, \forall i \in {\mathcal {I}} , \;\; {\tilde{\mathbf{{\mathcal {B}}} }}^*(x) = {\tilde{V}}{\tilde{R}}{\tilde{V}}^{T}\right\} . \end{aligned}$$

#### Proof

Let *Q* be specified in Theorem 2.6. Since ${{\,\mathrm{range}\,}}(V) = {{\,\mathrm{range}\,}}(Q{\tilde{V}})$, see Lemma 3.8, we assume w.l.g. that *V* in (3.19) satisfies $V = Q{\tilde{V}}$. Let $(x,{\tilde{R}})$ be feasible for (3.20). Define $X := \sum _{i=1}^{d}B_{i}x_{i}$. We show the equivalence in the following order: 
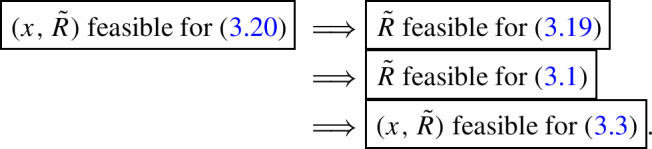
 Since $Q^{T}( \sum _{i=1}^{d}B_{i}x_{i})Q = {\tilde{V}}{\tilde{R}}{\tilde{V}}^{T} = Q^{T}V{\tilde{R}}V^{T}Q,$ we have $\sum _{i=1}^{d}B_{i}x_{i} = V{\tilde{R}}V^{T}$. Using the feasibility and (2.13), it holds that

$$\begin{aligned} \langle A_{i}, V{\tilde{R}}V^{T} \rangle = \left\langle A_{i}, \sum _{j=1}^{d}B_{j}x_{j}\right\rangle = \sum _{j=1}^{d} A_{i,j}x_{j} = b_{i}, \forall i \in {\mathcal {I}}. \end{aligned}$$

Thus all the linear constraints in (3.19) are satisfied, and ${\tilde{R}} \succeq 0$ is feasible for (3.19). By assumption, ${\tilde{R}}$ is also feasible for (3.1). Thus the constraints $\langle A_{i}, V{\tilde{R}}V^{T} \rangle = b_{i}, \forall i \notin {\mathcal {I}}$ are satisfied as well. This shows that $(x,{\tilde{R}})$ is feasible for (3.3). $\square$

To obtain a formulation for the facially and symmetry reduced program (3.3) in variable ${\tilde{R}}$ only, we can replace *x* in terms of ${\tilde{R}}$ using the constraint ${\tilde{\mathbf{{\mathcal {B}}} }}^*(x) = {\tilde{V}}{\tilde{R}}{\tilde{V}}^{T}$. This substitution can be done easily by rewriting the constraints as

$$\begin{aligned} b_{i} = \langle A_{i}, X \rangle = \langle Q^{T}A_{i}Q, Q^{T}XQ \rangle = \langle Q^{T}A_{i}Q, {\tilde{\mathbf{{\mathcal {B}}} }}^*(x) \rangle = \langle Q^{T}A_{i}Q, {\tilde{V}}{\tilde{R}}{\tilde{V}}^{T} \rangle . \end{aligned}$$

The objective can be similarly changed. This method, however, does not work for **DNN** relaxations. This difficulty can be resolved as follows.

#### Theorem 3.22

Consider the facially and symmetry reduced relaxation (3.3) with nonnegativity constraints,

3.21 $$\begin{aligned} \begin{aligned}&\min \{ c^{T}x \;|\; {Ax=b}, \;\; {\tilde{\mathbf{{\mathcal {B}}} }}^*(x) = {\tilde{V}}{\tilde{R}}{\tilde{V}}^{T}, \; {\tilde{R}} \succeq 0, \; x \ge 0\}, \end{aligned} \end{aligned}$$

where ${\tilde{R}} = {{\,\mathrm{{Blkdiag}}\,}}({\tilde{R}}_{1},\ldots ,{\tilde{R}}_{t})$. Equate *x* with

$$\begin{aligned} x\leftarrow f({\tilde{R}}) ={{{\,\mathrm{{Diag}}\,}}(w)^{-1}}\mathbf{{\mathcal {B}}} (V{\tilde{R}}V^{T}), \end{aligned}$$

where

$$\begin{aligned} w = (\langle B_{i}, B_{i} \rangle )_{i} \in {\mathbb {R}}^{d},\, V = Q{\tilde{V}}, \end{aligned}$$

and *Q* is specified in Theorem 2.6. Then (3.21) is equivalent to

3.22 $$\begin{aligned} \min \big \{ c^T\!f({\tilde{R}}) \;|\; {Af({\tilde{R}})=b}, \;\; {\tilde{R}} \succeq 0, \; f({\tilde{R}}) \ge 0\big \}, \end{aligned}$$

where the diagonal blocks in the block diagonal matrix ${\tilde{R}}$ in (3.22) that correspond to the repeating blocks in $Q^{T}A_{{{\mathcal {G}}} }Q$ are required to be equal.

#### Proof

If $(x,{\tilde{R}})$ is feasible for (3.21), then ${\mathbf{{\mathcal {B}}} ^*}(x) = V{\tilde{R}}V^{T}$. As $w>0$ and $\mathbf{{\mathcal {B}}} {\mathbf{{\mathcal {B}}} ^*}= {{\,\mathrm{{Diag}}\,}}(w)$, we have $x = f({\tilde{R}})$ and thus ${\tilde{R}}$ is feasible for (3.22).

Let ${\tilde{R}}$ be feasible for (3.22). Since ${\tilde{V}}{\tilde{R}}{\tilde{V}}^{T}$ is a block-diagonal matrix in the algebra $Q^{T}A_{{{\mathcal {G}}} }Q$, we have $V{\tilde{R}}V^{T} = Q{\tilde{V}}{\tilde{R}}{\tilde{V}}^{T}Q^{T} \in A_{{{\mathcal {G}}} }$. It follows from Theorem 2.6 that there exists a unique *x* such that $V{\tilde{R}}V^{T} = {\mathbf{{\mathcal {B}}} ^*}(x)$. Then we must have $x = f({\tilde{R}})$ and thus $(x,{\tilde{R}})$ is feasible for (3.21). $\square$

For the Hamming scheme, we have an explicit expression for the orthogonal matrix *Q* used in Theorem 3.22, see Example 2.5 and Sect. 5.1. In general, we do not know the corresponding orthogonal matrix explicitly. In Sect. 5.2, we use the heuristics from [15] to compute a block diagonalization of $A_{{\mathcal {G}}}$. In this case, the equivalence in Theorem 3.22 may not be true, and (3.22) may be weaker than (3.21). However our computational results indicate that all the bounds remain the same, see Table 7 below.

## The alternating direction method of multipliers, **ADMM**

It is well known that interior-point methods do not scale well for **SDP**. Moreover, they have great difficulty with handling additional cutting planes such as nonnegativity constraints. In particular, solving the doubly nonnegative relaxation, **DNN**, using interior-point methods is extremely difficult. The alternating direction method of multipliers is a first-order method for convex problems developed in the 1970s, and rediscovered recently. This method decomposes an optimization problem into subproblems that may be easier to solve. In particular, it is extremely successful for splittings with two cones. This feature makes the **ADMM** well suited for our large-scaled **DNN** problems. For state of the art in theory and applications of the **ADMM**, we refer the interested readers to [8].

Oliveira et al. [44] propose a version of the **ADMM** for solving an **SDP** relaxation for the Quadratic Assignment Problem (**QAP** ). Their computational experiments show that the proposed variant of the **ADMM** exhibits remarkable robustness, efficiency, and even provides improved bounds.

### Augmented Lagrangian

We modify the approach from [44] for solving our **SR** and **FR** reduced **DNN** relaxation (3.3). We have a greatly simplified structure as we applied **SR** to the **SDP** relaxation, and we were then able to move the nonnegativity constraints to a simple vector $x\ge 0$ contraint. We in particular obtain a more efficient approach for solving the *x*-subproblem.

Let ${\tilde{V}} = {{\,\mathrm{{Blkdiag}}\,}}({\tilde{V}}_{1},\ldots ,{\tilde{V}}_{t})$ and ${\tilde{R}} = {{\,\mathrm{{Blkdiag}}\,}}({\tilde{R}}_{1},\ldots ,{\tilde{R}}_{t})$. The augmented Lagrangian of (3.3) corresponding to the linear constraints ${\tilde{\mathbf{{\mathcal {B}}} }}^*(x) = {\tilde{V}}{\tilde{R}}{\tilde{V}}^{T}$ is given by:

$$\begin{aligned} {{{\mathcal {L}}} }(x,{\tilde{R}},{\tilde{Z}}) = \langle {\tilde{C}}, {\tilde{\mathbf{{\mathcal {B}}} }}^*(x)\rangle + \langle {\tilde{Z}}, {\tilde{\mathbf{{\mathcal {B}}} }}^*(x) - {\tilde{V}} {\tilde{R}}{\tilde{V}}^{T} \rangle + \frac{\beta }{2} ||{\tilde{\mathbf{{\mathcal {B}}} }}^*(x) - {\tilde{V}}{\tilde{R}}{\tilde{V}}^{T}||^{2}, \end{aligned}$$

where, see (2.12), ${\tilde{C}} = Q^{T}C Q$ is a block-diagonal matrix as $C \in A_{{\mathcal {G}}}$, ${\tilde{Z}}$ is also in block-diagonal form, and $\beta >0$ is the penalty parameter.

The alternating direction method of multipliers, **ADMM**, uses the *augmented Lagrangian*, ${{{\mathcal {L}}} }(x,{\tilde{R}},{\tilde{Z}})$, and essentially solves the max-min problem

$$\begin{aligned} \max _{{\tilde{Z}}} \min _{x\in P,{\tilde{R}} \succeq 0}{{{\mathcal {L}}} }(x,{\tilde{R}},{\tilde{Z}}), \end{aligned}$$

where *P* is a simple polyhedral set of constraints on *x*, e.g., linear constraints $Ax=b$ and nonnegativity constraints, see (4.3) below. The advantage of the method is the simplifications obtained for the constraints by taking advantage of the splitting in the variables. We then find the following updates $(x_+,{\tilde{R}}_+,{\tilde{Z}}_+)$:

$$\begin{aligned} x_+&= \arg \min _{x \in P} {{{\mathcal {L}}} }(x,{\tilde{R}},{\tilde{Z}}), \\ {\tilde{R}}_+&= \arg \min _{{\tilde{R}} \succeq 0 } {{{\mathcal {L}}} }(x_+,{\tilde{R}},{\tilde{Z}}), \\ {\tilde{Z}}_+&= {\tilde{Z}} + \gamma \beta ({\tilde{\mathbf{{\mathcal {B}}} }}^*(x_+) - {\tilde{V}}{\tilde{R}}_+{\tilde{V}}^{T}). \end{aligned}$$

Here, $\gamma \in (0, \frac{1+\sqrt{5}}{2})$ is the step size for updating the dual variable ${\tilde{Z}}$. In the following sections we explain in details how to solve each subproblem.

### On solving the ${{\tilde{R}}}$-subproblem

The ${{\tilde{R}}}$-subproblem can be explicitly solved. We complete the square and get the equivalent problem

4.1 $$\begin{aligned} \begin{array}{cll} {{\tilde{R}}}_+ &{}= &{} \min \limits _{{{\tilde{R}}} \succeq 0} ||{\tilde{\mathbf{{\mathcal {B}}} }}^*(x) - {\tilde{V}}{{\tilde{R}}}{\tilde{V}}^{T} + \frac{1}{\beta } {\tilde{Z}}||^{2} \\ &{}= &{} \min \limits _{{{\tilde{R}}} \succeq 0} ||{{\tilde{R}}} - {\tilde{V}}^{T}({\tilde{\mathbf{{\mathcal {B}}} }}^*(x)+ \frac{1}{\beta } {\tilde{Z}}){\tilde{V}}||^{2} \\ &{}= &{} \sum _{k=1}^{t} \min \limits _{{{\tilde{R}}}_{k} \succeq 0} ||\tilde{R}_{k} - \big ({\tilde{V}}^{T}({\tilde{\mathbf{{\mathcal {B}}} }}^*(x)+ \frac{1}{\beta } {\tilde{Z}}){\tilde{V}}\big )_{k}||^{2}. \end{array} \end{aligned}$$

Here, we normalize each block ${\tilde{V}}_k$ such that ${\tilde{V}}_k^T {\tilde{V}}_k=I$, and thus $\big ({\tilde{V}}^{T}({\tilde{\mathbf{{\mathcal {B}}} }}^*(x)+ \frac{1}{\beta } {\tilde{Z}}){\tilde{V}}\big )_{k}$ is the *k*-th block of ${\tilde{V}}^{T}({\tilde{\mathbf{{\mathcal {B}}} }}^*(x)+ \frac{1}{\beta } {\tilde{Z}}){\tilde{V}}$ corresponding to ${{\tilde{R}}}_{k}$. So we only need to solve *k* small problems whose optimal solutions are

$$\begin{aligned} {{\tilde{R}}}_{k} = {\mathcal {P}}_{{\mathbb {S}}_+}\left( {\tilde{V}}^{T}({\tilde{\mathbf{{\mathcal {B}}} }}^*(x)+ \frac{1}{\beta } {\tilde{Z}}){\tilde{V}}\right) _{k}, \quad k=1,\ldots ,t, \end{aligned}$$

where ${\mathcal {P}}_{{\mathbb {S}}_+}(M)$ is the projection onto the cone of positive semidefinite matrices.

### On solving the *x*-subproblem

For the *x*-subproblem, we have

4.2 $$\begin{aligned} x_+ = \arg \min \limits _{x \in P} \left\| {\tilde{\mathbf{{\mathcal {B}}} }}^*(x) - {\tilde{V}}{\tilde{R}}{\tilde{V}}^{T} + \frac{{\tilde{C}}+ {\tilde{Z}}}{\beta }\right\| ^{2}. \end{aligned}$$

For many combinatorial optimization problems, some of the constraints $Ax=b$ in (2.14) become redundant after **FR** of their semidefinite programming relaxations, see Corollary 3.21. Thus, the set *P* often collapses to a simple set. This often leads to an analytic solution for the *x*-subproblem; e.g., this happens for the quadratic assignment, graph partitioning, vertex separator, and shortest path problems.

For some interesting applications, the *x*-subproblem is equivalent to the following special case of the weighted, relaxed, quadratic knapsack problem:

4.3 $$\begin{aligned} \begin{array}{cll} \min _{x} &{} \frac{1}{2}||{{{\mathcal {T}}} } ^*(x)- Y ||^{2} \\ \text {s.t.} &{} x\in P:= \{x \,|\, w^{T}x = c,\, x\ge 0\}, \end{array} \end{aligned}$$

where *Y* is a given matrix and ${{{\mathcal {T}}} } ^*(x) = \sum _{i=1}^{q} x_{i}T_{i}$ for some given symmetric matrices $T_{i}$. The problem (4.3) is a projection onto the weighted simplex. We consider the following assumption on a linear transformation ${{{\mathcal {T}}} }: {\mathbb {S}}^n\rightarrow {\mathbb {R}}^q$ and its adjoint.

#### Assumption 4.1

The linear transformation ${{{\mathcal {T}}} }: {\mathbb {S}}^n\rightarrow {\mathbb {R}}^q$ in (4.3) satisfies

$$\begin{aligned} {{{\mathcal {T}}} } ({{{\mathcal {T}}} } ^*(x)) = {{\,\mathrm{{Diag}}\,}}(w)x, \, \forall x\in {\mathbb {R}}^q,\text { for some } w>0. \end{aligned}$$

#### Lemma 4.2

Suppose that the linear transformation ${{\mathcal {T}}}$ satisfies Assumption 4.1, and that (4.3) is feasible. Then the projection problem (4.3) can be solved efficiently (explicitly) using Algorithm 4.3.

#### Proof

The Lagrangian function of the problem is

$$\begin{aligned} \frac{1}{2}|| {{{\mathcal {T}}} } ^*(x) - {Y}||^{2} - \tau (w^{T}x - c) - \lambda ^{T}x, \end{aligned}$$

where $\tau \in {\mathbb {R}}$ and $\lambda \in {\mathbb {R}}_+^{q}$ are the Lagrangian multipliers. The KKT optimality conditions for the problem are given by

$$\begin{aligned} \begin{array}{rl} {{{\mathcal {T}}} } ({{{\mathcal {T}}} } ^*(x)) - {{{\mathcal {T}}} } ({Y}) - \tau w - \lambda &{}= 0, \\ x &{} \ge 0, \\ \lambda &{} \ge 0, \\ \lambda ^{T}x &{} = 0, \\ w^{T}x &{}= c. \end{array} \end{aligned}$$

Note that ${{\,\mathrm{{Diag}}\,}}(w)$ is the matrix representation of ${{\mathcal {T}}} \circ {{\mathcal {T}}} ^*$. This means that $\langle T_i,T_j \rangle =0, \forall i\ne j$, and we can simplify the first condition.^6^ This yields

$$\begin{aligned} x_{i} = w_{i}^{-1}({{{\mathcal {T}}} } ({Y}))_{i} + \tau + w_{i}^{-1}\lambda _{i}. \end{aligned}$$

Define the data vector $y := {{{\mathcal {T}}} } ({Y})$. The complementary slackness $\lambda ^{T}x = 0$ implies that if $x_{i} > 0$, then $\lambda _{i} = 0$ and $x_{i} = w_{i}^{-1}y_{i} + \tau$. If $x_{i} = 0$, then $w_{i}^{-1}y_{i} + \tau = -w_{i}^{-1}\lambda _{i} \le 0$. Thus the zero and positive entries of the optimal solution *x* correspond to the smaller than $-\tau$ and the larger than $-\tau$ entries of $(w_{i}^{-1}y_{i})_{i=1}^{q}$, respectively.

Let us assume, without loss of generality, that $(w_{i}^{-1}y_{i})_{i=1}^{q},x$ are sorted in non-increasing order:

$$\begin{aligned} \frac{y_{1}}{w_{1}} \ge \ldots \ge \frac{y_{k}}{w_{k}} \ge \frac{y_{k+1}}{w_{k+1}} \ge \ldots \ge \frac{y_{q}}{w_{q}}, \quad x_{1} \ge \ldots \ge x_{k} > x_{k+1} = \ldots =x_{q} = 0. \end{aligned}$$

The condition $w^{T}x = c$ implies that

$$\begin{aligned} w^{T}x = \sum _{i=1}^{k}w_{i}\left( \frac{y_{i}}{w_{i}} + \tau \right) = \sum _{i=1}^{k}y_{i} + \tau \sum _{i=1}^{k}w_{i} = c, \end{aligned}$$

and thus

$$\begin{aligned} \tau = \frac{c- \sum _{i=1}^{k}y_{i}}{\sum _{i=1}^{k}w_{i}}. \end{aligned}$$

Therefore, one can solve the problem by simple inspection once *k* is known. The following algorithm finds an optimal solution *x* to the problem (4.3). The correctness of the algorithm is then similar to the projection onto the (unweighted) simplex problem, see [10, 11]. $\square$


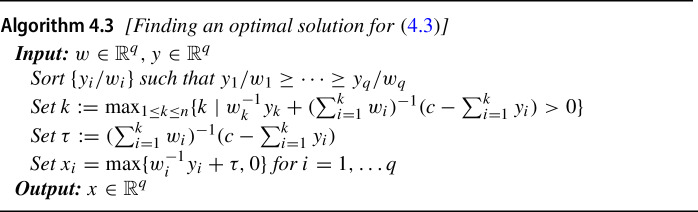


In our examples, see Sects. 5.1 and 5.2, the *x*-subproblem (4.2) satisfies Assumption 4.1. Moreover, we have the following lemma. We remind the reader that *J* denotes the matrix of all ones.

#### Lemma 4.3

The *x*-subproblem (4.2) satisfies Assumption 4.1, if

$$\begin{aligned} P = \{x \in {\mathbb {R}}^{q} \; | \; \langle J, \mathbf{{\mathcal {B}}} ^*(x) \rangle = c , x \ge 0\}. \end{aligned}$$

#### Proof

It holds that

4.4 $$\begin{aligned} \big ({\tilde{B}}({\tilde{B}}^*(x))\big )_{i} = \left\langle {\tilde{B}}_{i} , \sum _{j=1}^q {\tilde{B}}_{j}x_{j} \right\rangle = \langle {\tilde{B}}_{i}, {\tilde{B}}_{i} x_{i} \rangle = {{\,\mathrm{{trace}}\,}}(Q^{T}B_{i}^{T}QQ^{T}B_{i}Q) x_{i} = w_{i} x_{i},\nonumber \\ \end{aligned}$$

where $w_i = {{\,\mathrm{{trace}}\,}}(B_{i}^{T}B_{i})$. Furthermore, $\langle J, \mathbf{{\mathcal {B}}} ^*(x) \rangle = w^{T}x$ with $w = (w_{i}) \in {\mathbb {R}}^{q}$. Thus we set ${{\mathcal {T}}} = \mathbf{{\mathcal {B}}}$ and note that ${{{\mathcal {T}}} } ({{{\mathcal {T}}} } ^*(x)) = {{\,\mathrm{{Diag}}\,}}(w)x$. $\square$

## Numerical results

We now demonstrate the efficiency of our new approach on two classes of problems: the quadratic assignment problem, **QAP**, and several types of graph partitioning problem, **GP**.

Our tests were on: Dell PowerEdge M630 computer; two Intel Xeon E5-2637v3 4-core 3.5 GHz (Haswell) CPU; 64GB memory; linux. The interior point solver was Mosek, see [1]. We had to use a different computer to accommodate some of the larger problems when using an interior point approach, see description of Table 4.

We report only the ADMM solver time, since the preprocessing time is very small in comparison with the ADMM part. In particular, we find exposing vectors explicitly in all our examples. Further, for most of the test instances we know generators of automorphism groups as well as the orthogonal matrix *Q*. For instances for which we need to find generators e.g., the instances in Table 3 the preprocessing time is less than one second.

The stopping conditions and tolerances are outlined at the start of Sect. 5.1.3, in Definition 5.10. Our results include *huge* problems of sizes up to $n=512$ for the **QAP**, yielding of the order $n^2$ **SDP** constraints and $n^4$ nonnegativity constraints.^7^

### The quadratic assignment problem, **QAP**

#### Background for the **QAP**

The Quadratic Assignment Problem was introduced in 1957 by Koopmans and Beckmann as a model for location problems that take into account the linear cost of placing a new facility on a certain site, plus the quadratic cost arising from the product of the flow between facilities and distances between sites. The **QAP** contains the traveling salesman problem as a special case and is therefore NP-hard in the strong sense. It is generally considered to be one of the *hardest* of the NP-hard problems.

Let $A,B\in {\mathbb {S}}^n$, and let $\Pi _n$ be the set of $n \times n$ permutation matrices. The **QAP** (with the linear term with appropriate *C* in brackets) can be stated as follows:

$$\begin{aligned} \min \limits _{X \in \Pi _n} {{\,\mathrm{{trace}}\,}}(AX^TBX) \quad (+{{\,\mathrm{{trace}}\,}}(X^TC)). \end{aligned}$$

The **QAP** is extremely difficult to solve to optimality, e.g., problems with $n\ge 30$ are still considered hard. It is well known that **SDP** relaxations provide strong bounds, see e.g., [15, 69]. However even for sizes $n\ge 15$, it is difficult to solve the resulting **SDP** relaxation by interior point methods if one cannot exploit special structure such as symmetry. Solving the **DNN** relaxation is significantly more difficult.

Here, we first consider the **DNN** relaxation for the **QAP** from Povh and Rendl [50], i.e.,

5.1 $$\begin{aligned} \begin{array}{rl} \min &{} {{\,\mathrm{{trace}}\,}}(A\otimes B) Y \\ \text {s.t.} &{} \langle J_{n^{2}},Y \rangle = n^{2} \\ &{} \langle I_n \otimes (J_n-I_n) + (J_n-I_n) \otimes I_n,Y \rangle = 0 \\ &{} \langle I_n \otimes E_{ii}, Y \rangle = 1, \forall i = 1,\ldots ,n\\ &{} \langle E_{ii} \otimes I_n, Y \rangle = 1, \forall i = 1,\ldots ,n\\ &{} Y \succeq 0, Y \ge 0, \quad (Y\in {\textbf {DNN}}) \end{array} \end{aligned}$$

where and $E_{ii}=u_iu_i^T$, where $u_i\in {\mathbb {R}}^n$ is *i*-th unit vector. The authors in [15, Theorem 7.1] show that one can take

5.2 $$\begin{aligned} {{\mathcal {A}}}_{{{\mathcal {G}}} } = {{{\mathcal {A}}}} _{{{{\,\mathrm{{aut}}\,}}}(A)} \otimes {{{\mathcal {A}}}} _{{{{\,\mathrm{{aut}}\,}}}(B)}, \end{aligned}$$

where ${{{\,\mathrm{{aut}}\,}}}(A):= \{ P \in \Pi _n: AP=PA \}$ is the automorphism group of *A*.

##### Remark 5.1

The **DNN** relaxation (5.1) is known to be theoretically equivalent, yielding the same optimal value, to the **DNN** relaxation denoted (QAP$_{R3}$) in Zhao et al. [69]. The constraints $\langle I_n \otimes (J_n-I_n) + (J_n-I_n) \otimes I_n,Y \rangle = 0$ are generally called the *gangster constraints*, see Lemma 5.4. The third and fourth lines of constraints in (5.1) arise from the row and column sum constraints.

Recall that ${{\,\mathrm{{svec}}\,}}$ is the linear transformation that vectorizes symmetric matrices, [69]. We define ${{\,\mathrm{{gsvec}}\,}}$ *to do this vectorization of symmetric matrices while ignoring the elements set to zero by the gangster constraints.* Then we can eliminate the gangster constraints completely and replace the **DNN** constraints to get the equivalent problem to (5.1):

5.3 $$\begin{aligned} \begin{array}{rll} \min &{} {{\,\mathrm{{gsvec}}\,}}(A\otimes B)^T y \\ \text {s.t.} &{} {{\,\mathrm{{gsvec}}\,}}(J_{n^{2}})^Ty = n^{2} \\ &{} {{\,\mathrm{{gsvec}}\,}}( I_n \otimes E_{ii})^T y = 1, &{} \forall i = 1,\ldots ,n\\ &{} {{\,\mathrm{{gsvec}}\,}}( E_{ii} \otimes I_n)^T y = 1,&{} \forall i = 1,\ldots ,n\\ &{} {{\,\mathrm{{gsvec}}\,}}^*(y) \succeq 0,\, y \ge 0. \end{array} \end{aligned}$$

This form is now similar to our final **SR** reduced form before **FR**, see (2.14); and this emphasizes that the **DNN** can be represented in a split form.

In the following lemma we derive the null space of the feasible solutions of (5.1), see also Corollary 2.2.7 in [60].

##### Lemma 5.2

Let $U := \frac{1}{\sqrt{n}}(nI-J) \in {\mathbb {R}}^{n \times n}$, and let *Y* be in the relative interior of the feasible set of (5.1). Then

$$\begin{aligned} {{\,\mathrm{null}\,}}(Y) = {{\,\mathrm{range}\,}}\left( \begin{bmatrix} U \otimes e_{n}&e_{n} \otimes U \end{bmatrix}\right) . \end{aligned}$$

##### Proof

Let $X \in \Pi _n$. Then $Xe_n = X^{T}e_n = e_n$, and thus

$$\begin{aligned} \begin{array}{lr} (U \otimes e_{n})^T\text {vec}(X) = U^{T}e_n = 0,\\ (e_{n} \otimes U)^{T} \text {vec}(X) = U^{T}e_n = 0. \end{array} \end{aligned}$$

Thus $${{\,\mathrm{range}\,}}\left( \begin{bmatrix} U \otimes e_{n}&e_{n} \otimes U \end{bmatrix}\right) \subseteq {{\,\mathrm{null}\,}}({\hat{Y}})$$, where

$$\begin{aligned} \hat{Y} = \frac{1}{n!} \sum \limits _{X\in \Pi _n} \mathrm{vec}(X)\mathrm{vec}(X)^T = \frac{1}{n^2}( J\otimes J ) + \frac{1}{n^2(n-1)}(nI-J)\otimes ( nI-J ). \end{aligned}$$

It is proven in [60] that $\hat{Y}$ is in the relative interior of the feasible set of (5.1). Recall that every matrix *Y* in the relative interior of a face has the same null space. This shows that $${{\,\mathrm{null}\,}}(Y) \supseteq {{\,\mathrm{range}\,}}\left( \begin{bmatrix} U \otimes e_{n}&e_{n} \otimes U \end{bmatrix}\right) $$.

It remains to show that

$$\begin{aligned} \dim \left( {{\,\mathrm{range}\,}}\left( \begin{bmatrix} U\otimes e_{n}&e_{n} \otimes U \end{bmatrix} \right) \right) = 2(n-1). \end{aligned}$$

To see this, we choose the square submatrix of size $2n-1$ associated to

$$\begin{aligned}&\text {rows: } \{kn \;|\; k = 1,\ldots ,n-1\} \cup \{ n(n-1)+1,\ldots , n^2-1\}; \\&\quad \text {cols: } \{1,\ldots ,n-1\} \cup \{n+1,\ldots ,2n\}. \end{aligned}$$

It has the form

$$\begin{aligned} \frac{1}{\sqrt{n}}(nI-J) \in {\mathcal {S}}^{2(n-1)}. \end{aligned}$$

This square submatrix clearly has rank $2(n-1)$, and thus the statement follows. $\square$

Let us now derive an exposing vector of the **SDP** relaxation (5.1) ignoring the nonnegativity, as we have shown we can add the nonnegativity on after the reductions.

##### Lemma 5.3

Consider (5.1) without the nonnegativity constraints. Then

5.4 $$\begin{aligned} \begin{array}{rl} W = I_n \otimes nJ_n + J_n \otimes (nI_n-2J_n) \in {{\mathcal {A}}}_{{{\mathcal {G}}} } \subseteq {\mathcal {S}}^{n^{2}}_+, \end{array} \end{aligned}$$

and is an exposing vector of rank $2 (n-1)$ in ${{\mathcal {A}}}_{{{\mathcal {G}}} }$.

##### Proof

Let *U* be defined as in Lemma 5.2. Using the properties of the Kronecker product, we have

5.5 $$\begin{aligned} \begin{array}{rl} 0\preceq W=&\begin{bmatrix} U\otimes e_{n}&e_{n} \otimes U \end{bmatrix} \begin{bmatrix} U\otimes e_{n} &{} e_{n} \otimes U \end{bmatrix} ^{T} \\ = &{} (UU^{T}) \otimes J + J \otimes (UU^{T}) \\ = &{} (nI-J) \otimes J + J \otimes (nI-J) \\ = &{} I \otimes nJ + J \otimes (nI-2J), \end{array} \end{aligned}$$

as $UU^{T} = nI-J$. From Lemma 5.2, we have *W* is an exposing vector of rank $2(n-1)$. Let *P* be any permutation matrix of order *n*. Then $P^{T}(UU^{T})P = UU^{T}$ by construction. We now have $(P_1\otimes P_2)^{T}W(P_1\otimes P_2) = W$, for any $P_1,P_2\in \Pi _n$; and thus $W \in {{\mathcal {A}}}_{{{\mathcal {G}}} }$. $\square$

In the rest of this section we show how to do **FR** for the symmetry reduced program of (5.1). We continue to add on nonnegativity constraints to **SDP** relaxations as discussed above. The facially reduced formulation of (5.1) is also presented in [60]. We state it here for later use.

##### Lemma 5.4

( [60]) The facially reduced program of the *doubly nonnegative,* ***DNN*** (5.1) is given by

5.6 $$\begin{aligned} \begin{array}{cl} \min &{} \left\langle \left( V^{T} \left( A\otimes B \right) V \right) ,R\right\rangle \\ \text {s.t.} &{} \left\langle V^{T}JV ,R \right\rangle = n^2 \\ &{} {{\mathcal {G}}} (VRV^{T}) = 0 \\ &{} VRV^{T} \ge 0 \\ &{} R \in {\mathcal {S}}^{(n-1)^2+1}_+, \end{array} \end{aligned}$$

where, by abuse of notation, ${{\mathcal {G}}} : {\mathcal {S}}^{n^2} \rightarrow {\mathcal {S}}^{n^2}$ is a linear operator defined by ${{\mathcal {G}}} (Y) := (J- (I\otimes (J-I) + (J-I)\otimes I) ) \circ Y$^8^, and the columns of $V \in {\mathbb {R}}^{n^2 \times (n-1)^2+1}$ form a basis of the null space of *W*, see Lemma 5.3.

Note that the constraints $\langle I \otimes E_{ii}, Y \rangle = 1$ and $\langle E_{ii} \otimes I, Y \rangle = 1$ have become redundant after **FR** in (5.6).

We now discuss the symmetry reduced program. The symmetry reduced formulation of (5.1) is studied in [16]. We assume that the the automorphism group of the matrix *A* is non-trivial. To simplify the presentation, we assume

$$\begin{aligned} A = \sum _{i=0}^{d}a_{i}A_{i}, \end{aligned}$$

where $\{A_{0},\ldots ,A_{d}\}$ is the basis of the commutant of the automorphism group of *A*. For instance the matrices $A_i$ ($i=0,1,\ldots ,d$) may form a basis of the Bose-Mesner algebra of the Hamming scheme, see Example 2.5. Further, we assume from now on that $A_0$ is a diagonal matrix, which is the case for the Bose-Mesner algebra of the Hamming scheme. Here, we do not assume any structure in *B*. However the theory applies also when *B* has some symmetry structure and/or $A_0$ is not diagonal; see our numerical tests for the minimum cut problem in Sect. 5.2, below.

If the **SDP**  (5.1) has an optimal solution $Y \in {\mathcal {S}}^{n^2}_+$, then it has an optimal solution of the form $Y =\sum _{i=0}^{d} A_{i} \otimes Y_{i}$ for some matrix variables $Y_{0},\ldots ,Y_{d} \in {\mathbb {R}}^{n \times n}$, see (5.2) and Sect. 2.2. We write these matrix variables in a more compact way as $y = (\text {vec}(Y_{0}),\ldots ,\text {vec}(Y_{d}))$, if necessary. Denote by ${\tilde{\mathbf{{\mathcal {B}}} }}^*_{k}(y) \in {\mathcal {S}}^{n_{k}}_+$ the *k*-th block of the block-diagonal matrix

5.7 $$\begin{aligned} {\tilde{\mathbf{{\mathcal {B}}} }}^*(y) :=(Q \otimes I)^{T} Y (Q \otimes I) = \sum _{i=0}^{d}(Q^{T}A_{i}Q) \otimes Y_{i}, \end{aligned}$$

where *Q* is the orthogonal matrix block-diagonalizing $A_i$ ($i=0,\ldots , d$).

##### Lemma 5.5

The symmetry reduced program of the **DNN** relaxation (5.1) is given by

5.8 $$\begin{aligned} \begin{array}{cl} \min &{} \sum _{i=0}^{d} a_{i} {{\,\mathrm{{trace}}\,}}(A_{i}A_{i}) {{\,\mathrm{{trace}}\,}}(BY_{i}) \\ \text {s.t.} &{} \sum _{i=0}^{d} {{\,\mathrm{{trace}}\,}}(J A_{i}) {{\,\mathrm{{trace}}\,}}(J Y_{i} ) = n^2 \\ &{} {{\,\mathrm{{offDiag}}\,}}(Y_{0}) = 0 \\ &{} {{\,\mathrm{{diag}}\,}}(Y_{i}) = 0,\, i = 1,\ldots ,d \\ &{} {{\,\mathrm{{diag}}\,}}(Y_{0}) = \frac{1}{n}e_n \\ &{} Y_{j} \ge 0, j = 0,\ldots ,d \\ &{} {\tilde{\mathbf{{\mathcal {B}}} }}^*_{k}(y) \in {\mathcal {S}}^{n_{k}}_+, k = 1,\ldots ,t, \end{array} \end{aligned}$$

where ${\tilde{\mathbf{{\mathcal {B}}} }}^*_{k}(y)$ is the *k*-th block from (5.7), and ${{\,\mathrm{{offDiag}}\,}}(Y_0)=0$ is the linear constraints that the off-diagonal elements are zero.

##### Proof

See e.g., [15, 60]. $$\square $$

It remains to facially reduce the symmetry reduced program (5.8). Note that $W \in A_{{{\mathcal {G}}} }$ can be written as $W = \sum _{i=0}^{d} A_{i} \otimes W_{i}$, for some matrices $W_{0},\ldots ,W_{d} \in {\mathbb {R}}^{n \times n}$. Theorem 3.6 shows that the block-diagonal matrix

5.9 $$\begin{aligned} {\widetilde{W}}:=(Q\otimes I)^{T}W(Q \otimes I) = \sum _{i=0}^{d} (Q^{T}A_{i}Q) \otimes W_{i} \end{aligned}$$

is an exposing vector of the symmetry reduced program (5.8). Further, we denote by ${\widetilde{W}}_k$ ($k=1,\ldots , t$) the *k*-th block of ${\widetilde{W}}$. Let us illustrate this with Example 5.6.

##### Example 5.6

Consider Example 2.5, where $A_i$ ($i=0,\ldots ,d$) form a basis of the Bose-Mesner algebra of the Hamming scheme. Then, the exposing vector $W \in {\mathcal {S}}^{n^{2}}_+$ defined in Lemma 5.3 can be written as $W = \sum _{i=0}^{d} A_{i} \otimes W_{i}$, where

5.10 $$\begin{aligned} W_{0} = (n-2)J + nI \text { and } W_{i} = nI_{n} - 2J \text { for } i = 1,\ldots ,d. \end{aligned}$$

Let ${\widetilde{W}}_{k} \in {\mathcal {S}}^{n}$ be the *k*-th block of ${\widetilde{W}}$, see (5.9). Then there are $d+1$ distinct blocks given by ${\widetilde{W}}_{k} = \sum _{i=0}^{d}p_{i,k}W_{i} \in {\mathcal {S}}^{n}$ for $k = 0,\ldots ,d$, where $p_{i,k}$ are elements in the character table *P* of the Hamming scheme, see Example 2.5. Using the fact that $Pe = (n,0,\ldots ,0)^{T}$ and $p_{1,k} = 1$, for every $k = 0,\ldots ,d$, we have

5.11 $$\begin{aligned} {\widetilde{W}}_{0} = n^{2}I - nJ \text { and } {\widetilde{W}}_{k} = nJ \text { for } k = 1,\ldots ,d, \end{aligned}$$

and the matrices ${\tilde{V}}_{k}$, whose columns form a basis of the null space of ${\widetilde{W}}_{k} \in {\mathcal {S}}^{n}$, are given by

5.12 $$\begin{aligned} {\tilde{V}}_{0} = e_n \text { and } {\tilde{V}}_{k} = \begin{bmatrix} I_{n-1} \\ -e^T_{n-1} \end{bmatrix} \in {\mathbb {R}}^{n \times (n-1)} \text { for } k = 1,\ldots ,d. \end{aligned}$$

Similar results can be derived when one uses different groups. Now we are ready to present an **SDP** relaxation for the **QAP** that is both facially and symmetry reduced.

##### Proposition 5.7

The facially reduced program of the symmetry reduced **DNN** relaxation (5.8) is given by

5.13 $$\begin{aligned} \begin{array}{cl} \min &{} \displaystyle \sum _{i=1}^{d} a_{i} {{\,\mathrm{{trace}}\,}}(A_{i}A_{i}) {{\,\mathrm{{trace}}\,}}(BY_{i}) \\ \text {s.t.} &{} \displaystyle \sum _{i=0}^{d} {{\,\mathrm{{trace}}\,}}(J A_{i}) {{\,\mathrm{{trace}}\,}}(J Y_{i} ) = n^2 \\ &{} {{\,\mathrm{{offDiag}}\,}}(Y_{0}) = 0 \\[1ex] &{} {{\,\mathrm{{diag}}\,}}(Y_{i}) = 0, i = 1,\ldots ,d \\[1ex] &{} Y_{j} \ge 0, j = 0,\ldots ,d \\ &{} {\tilde{\mathbf{{\mathcal {B}}} }}^*_{k}(y) = {\tilde{V}}_{k}{\tilde{R}}_{k}{\tilde{V}}_{k}^{T}, k = 1,\ldots ,t\\ &{} {\tilde{R}}_{k} \in {\mathcal {S}}^{n_{k}^\prime }_+, k = 1,\ldots ,t. \end{array} \end{aligned}$$

Here, the columns of ${\tilde{V}}_{k} \in {\mathbb {R}}^{n_k \times n_{k}^\prime }$ form a basis of the null space of the ${\widetilde{W}}_{k} \in {\mathcal {S}}^{n}$.

##### Proof

Applying Theorem 3.6 to the block-diagonal matrix $(Q\otimes I)^{T}W(Q \otimes I) = \sum _{i=0}^{d} (Q^{T}A_{i}Q) \otimes W_{i}$, the matrices ${\widetilde{W}}_{k}$ are the exposing vectors of the symmetry reduced program (5.8), and thus ${\widetilde{W}}_{k} {\tilde{\mathbf{{\mathcal {B}}} }}^*_{k}(y)= 0$ for every $k = 1,\ldots ,t$. This means that there exists a full column rank matrix ${\tilde{V}}_{k} \in {\mathbb {R}}^{n_k \times n_{k}^\prime }$ such that ${\tilde{\mathbf{{\mathcal {B}}} }}^*_{k}(y) = {\tilde{V}}_{k}{\tilde{R}}_{k}{\tilde{V}}_{k}^{T}$, where ${\tilde{R}}_{k} \in {\mathcal {S}}^{n_{k}^\prime }_+$ for every $k = 1,\ldots ,t$. Finally, we apply Corollary 3.21 to remove redundant constraints, see also Lemma 5.4. This yields the formulation (5.13). $\square$

Note that in the case that the basis elements $A_i$ ($i=0,\ldots , d$) belong to the Hamming scheme, see Example 5.6, it follows that $t=d+1$ in the above Proposition 5.7.

#### On solving **QAP** with **ADMM** 

Now we discuss how to use **ADMM** to solve the **DNN** relaxation (5.13), for the particular case when $A_i$ ($i=0,1,\ldots ,d$) form a basis of the Bose-Mesner algebra of the Hamming scheme. We proceed as in Sect. 4, and exploit properties of the known algebra, see Example 2.5. Clearly, for any other algebra we can proceed in a similar way. We assume without loss of generality that all the matrices ${\tilde{V}}_{j}$ in this section have orthonormal columns.

First, we derive the equivalent reformulation of the **DNN** relaxation (5.13), by exploiting the following.

1. Since we remove the repeating blocks of positive semidefinite constraints, to apply **ADMM** we have to reformulate the **DNN** in such a way that Assumption 4.1 is satisfied. Let us first derive an expression for the objective function as follows.
  $$\begin{aligned} \begin{array}{rl} {{\,\mathrm{{trace}}\,}}((A \otimes B) Y) &{} = {{\,\mathrm{{trace}}\,}}\big ( (Q \otimes I)^{T}(\sum _{i=0}^{d}a_{i}A_{i} \otimes B) (Q \otimes I) (Q \otimes I)^{T}(\sum _{j=0}^{d} A_{j} \otimes Y_{j}) (Q \otimes I)\big ) \\ &{} = \displaystyle {{\,\mathrm{{trace}}\,}}\bigg ( \big ( \sum _{i=0}^{d}(Q^{T}a_{i}A_{i}Q) \otimes B \big ) \big ( \sum _{j=0}^{d} (Q^{T}A_{j}Q) \otimes Y_{j} \big ) \bigg ) \\ &{}= \displaystyle \sum _{k=0}^{d} \mu _{k} {{\,\mathrm{{trace}}\,}}\big ((\sum _{i=0}^{d} a_{i} p_{i,k}B) ( \sum _{j=0}^{d} p_{j,k}Y_{j}) \big ) \\ &{} = \displaystyle \sum _{k=0}^{d} \langle {\tilde{C}}_{k}, \sqrt{\mu _{k}} \sum _{i=0}^{d} p_{i,k}Y_{i} \rangle , \end{array} \end{aligned}$$
  where ${\tilde{C}}_{k} := \sqrt{\mu _{k}} (\sum _{i=0}^{d} a_{i} p_{i,k}) B$. Recall that $\mu = (\mu _{k}) \in {\mathbb {R}}^{d+1}$, with $\mu _k := {d \atopwithdelims ()k}(q-1)^{k}$. Then, we multiply the coupling constraints ${\tilde{\mathbf{{\mathcal {B}}} }}^*_{i}(y) = {\tilde{V}}_{i}{\tilde{R}}_{i}{\tilde{V}}_{i}^{T}$ by the square root of its multiplicities. Thus, for the Bose-Mesner algebra, we end up with $\sqrt{\mu _{j}}(\sum _{i=0}^{d}p_{i,j}Y_{i} - {\tilde{V}}_{j}{\tilde{R}}_{j}{\tilde{V}}_{j}^{T}) = 0$.
2. In many applications, it is not necessary to compute high-precision solutions, and the **ADMM** can be terminated at any iteration. Then, one can use the dual variable ${\tilde{Z}}_{j}$ from the current iteration to compute a valid lower bound, see Lemma 5.9. By adding redundant constraints, this lower bound is improved significantly when the **ADMM** is terminated with low-precision. Therefore we add the following redundant constraints
  5.14 $$\begin{aligned} Y_{0} = \frac{1}{n} I, \quad {{\,\mathrm{{trace}}\,}}({\tilde{R}}_{j}) = \sqrt{\mu _{j}} p_{0,j} \text { for } j = 0,\ldots ,d. \end{aligned}$$
  To see the redundancy of the last $d+1$ constraints above, we use the fact that the columns of ${\tilde{V}}_{j}$ are orthonormal, and that ${{\,\mathrm{{diag}}\,}}(Y_{i}) = 0, i=1,\ldots ,d$, to derive
  $$\begin{aligned} {{\,\mathrm{{trace}}\,}}({\tilde{R}}_{j}) = {{\,\mathrm{{trace}}\,}}( {\tilde{V}}_{j}{\tilde{R}}_{j}{\tilde{V}}_{j}^{T}) = {{\,\mathrm{{trace}}\,}}\sqrt{\mu _{j}}\left( \sum _{i=0}^{d}p_{i,j}Y_{i} \right) = \sqrt{\mu _{j}} p_{0,j}. \end{aligned}$$
  This technique can also be found in [26, 35, 44, 48].

We would like to emphasize that the techniques above are not restricted to the Bose-Mesner algebra of the Hamming scheme. Let us present our reformulated **DNN** relaxation for **ADMM** . Define

5.15 $$\begin{aligned} \begin{array}{l} {\mathcal {P}}:= \displaystyle \left\{ (Y_{0},\ldots ,Y_{d}) \;|\; \sum _{i=0}^{d} {d \atopwithdelims ()i}(q-1)^{i}q^{d} {{\,\mathrm{{trace}}\,}}(JY_{i}) = n^2,\; \right. \\ \left. \qquad \qquad \qquad \qquad Y_{0} =\displaystyle \frac{1}{n} I,\; {{\,\mathrm{{diag}}\,}}(Y_{i}) = 0, Y_{j} \ge 0,\; i = 1,\ldots ,d\right\} , \end{array} \end{aligned}$$

and

5.16 $$\begin{aligned} \mathcal {{\tilde{R}}}:= \{ ({\tilde{R}}_{0},\ldots ,{\tilde{R}}_{d}) \;|\; {{\,\mathrm{{trace}}\,}}({\tilde{R}}_{j}) = \sqrt{\mu _{j}} p_{0,j},\; {\tilde{R}}_{i} \in {\mathcal {S}}^{n}_+, \; i = 0,\ldots ,d\}.\qquad \end{aligned}$$

We obtain the following **DNN** relaxation for our **ADMM** .

5.17 $$\begin{aligned} \begin{array}{ccl} p^{*}:=&{} \min &{} \displaystyle \sum _{j=0}^{d} \langle {\tilde{C}}_{j}, \sqrt{\mu _{j}}\sum _{i=0}^{d} p_{i,j}Y_{i} \rangle \\ &{} \text {s.t.} &{} (Y_{0},\ldots ,Y_{d}) \in {\mathcal {P}} \\ &{} &{} ({\tilde{R}}_{0},\ldots ,{\tilde{R}}_{d}) \in {\mathcal {R}} \\ &{} &{} \displaystyle \sqrt{\mu _{j}}(\sum _{i=0}^{d}p_{i,j}Y_{i} - {\tilde{V}}_{j}{\tilde{R}}_{j}{\tilde{V}}_{j}^{T}) = 0, j = 0,\ldots ,d. \\ \end{array} \end{aligned}$$

The augmented Lagrangian is

$$\begin{aligned} \begin{array}{l} {{{\mathcal {L}}} }({\tilde{Y}},{\tilde{R}},{\tilde{Z}}):=\displaystyle \sum _{j=0}^{d} \Big ( \langle {\tilde{C}}_{j}, \sqrt{\mu _{j}}\sum _{i=0}^{d} p_{i,j}Y_{i} \rangle + \langle {\tilde{Z}}_{j}, \sqrt{\mu _{j}}(\sum _{i=0}^{d}p_{i,j}Y_{i} - {\tilde{V}}_{j}{\tilde{R}}_{j}{\tilde{V}}_{j}^{T}) \rangle \\ \qquad \qquad \qquad \qquad \qquad \qquad \qquad + \displaystyle \frac{\beta }{2} ||\sqrt{\mu _{j}}(\sum _{i=0}^{d}p_{i,j}Y_{i} - {\tilde{V}}_{j}{\tilde{R}}_{j}{\tilde{V}}_{j}^{T}) ||^{2} \Big ). \end{array} \end{aligned}$$

The *Y*-subproblem, the ${\tilde{R}}$-subproblem and the dual update are represented below.

1. The *Y*-subproblem:
  5.18 $$\begin{aligned} \begin{array}{cl} \min &{}\displaystyle \sum _{j=0}^{d}||\sqrt{\mu _{j}} \sum _{i=0}^{d}p_{i,j}Y_{i} - \sqrt{\mu _{j}} {\tilde{V}}_{j}{\tilde{R}}_{j}{\tilde{V}}_{j}^{T} + \frac{{\tilde{C}}_{j}+ {\tilde{Z}}_{j}}{\beta }||^{2} \\ \text {s.t.} &{} Y_{0} = \frac{1}{n}I\\ &{} {{\,\mathrm{{diag}}\,}}(Y_{i}) = 0, ~i = 1,\ldots ,d \\ &{} \displaystyle \sum _{i=0}^{d} {d \atopwithdelims ()i}(q-1)^{i}q^{d} {{\,\mathrm{{trace}}\,}}(JY_{i}) = n^2 \\ &{} Y_{i} \ge 0, ~i = 0,\ldots ,d. \end{array} \end{aligned}$$
2. The ${\tilde{R}}$-subproblems, for $j = 0,\ldots ,d$:
  5.19 $$\begin{aligned} \begin{array}{cl} \min &{}\displaystyle ||{\tilde{R}}_{j} - {\tilde{V}}_{j}^{T}(\sum _{i=0}^{d}p_{i,j}Y_{i} + \frac{{\tilde{Z}}_{j}}{\beta \sqrt{\mu _{j}}}){\tilde{V}}_{j}||^{2} \\ \text {s.t.} &{} {\tilde{R}}_{j} \in {\mathcal {S}}^{n_{j}^\prime }_+. \end{array} \end{aligned}$$
3. Update the dual variable:
  5.20 $$\begin{aligned} {\tilde{Z}}_{j} \leftarrow {\tilde{Z}}_{j} + \gamma \beta \sqrt{\mu _{j}} (\sum _{i=0}^{d}p_{i,j}Y_{i} - {\tilde{V}}_j{\tilde{R}}_j{\tilde{V}}_j^{T}), \quad j = 0,\ldots ,d. \end{aligned}$$

Clearly, the ${\tilde{R}}$-subproblems can be solved in the same way as (4.1). To see that the *Y*-subproblem can also be solved efficiently, let us show that it is a problem of the form (4.3), and thus satisfies Assumption 4.1.

Let $$\lambda _{j} = (p_{0,j},\ldots ,p_{d,j})^{T} $$,

$$\begin{aligned} y = \begin{bmatrix} \text {vec}(Y_{0})\\ \vdots \\ \text {vec}(Y_{d})\\ \end{bmatrix} \text { and } \hat{y} = \begin{bmatrix} \text {vec}\left( \sqrt{\mu _{0}} {{\tilde{V}}}_{0} {{\tilde{R}}}_{0}{{\tilde{V}}}_{0}^{T} - \frac{{\tilde{C}}_{0} + {{\tilde{Z}}}_{0}}{\beta }\right) \\ \vdots \\ \text {vec}\left( \sqrt{\mu _{d}} {{\tilde{V}}}_{d} {{\tilde{R}}}_{d} {{\tilde{V}}}_{d}^{T} - \frac{{\tilde{C}}_{d} + {{\tilde{Z}}}_{d}}{\beta }\right) \end{bmatrix}. \end{aligned}$$

Define the linear transformation ${{{\mathcal {T}}} } ^*: {\mathbb {R}}^{(d+1)n^2} \rightarrow {\mathbb {R}}^{(d+1)n^2}$ by

$$\begin{aligned} {{{\mathcal {T}}} } ^*(y) = \begin{bmatrix} \sqrt{\mu _{0}} \big ( \lambda _{0}^{T} \otimes I_{n^{2}} \big ) \\ \vdots \\ \sqrt{\mu _{d}} \big ( \lambda _{d}^{T} \otimes I_{n^{2}} \big ) \\ \end{bmatrix}y. \end{aligned}$$

##### Lemma 5.8

The *Y*-subproblem (5.18) is equivalent to the following projection to the weighted simplex problem

5.21 $$\begin{aligned} \begin{array}{cl} \min &{} ||{{{\mathcal {T}}} } ^*(y) - \hat{y}||^{2} \\ \text {s.t.} &{} y_{i} = 0, i \in {\mathcal {I}}\\ &{} w^{T}y = n^2 \\ &{} y \ge 0, \end{array} \end{aligned}$$

where $w := q^{d} (\mu \otimes e_{n^{2}}) \in {\mathbb {R}}^{(d+1)n^2}$, and ${\mathcal {I}}$ contains the indices of *y* associated to the off-diagonal entries of $Y_{0}$. Furthermore, the problem (5.21) satisfies Assumption 4.1.

##### Proof

One can verify that (5.18) and (5.21) are equivalent. Furthermore, it holds that

$$\begin{aligned} {{{\mathcal {T}}} } ({{{\mathcal {T}}} } ^*(y))= & {} \begin{bmatrix} \sqrt{\mu _{0}} \big ( \lambda _{0}^{T} \otimes I_{n^{2}} \big ) \\ \vdots \\ \sqrt{\mu _{d}} \big ( \lambda _{d}^{T} \otimes I_{n^{2}} \big ) \\ \end{bmatrix}^{T}\begin{bmatrix} \sqrt{\mu _{0}} \big ( \lambda _{0}^{T} \otimes I_{n^{2}} \big ) \\ \vdots \\ \sqrt{\mu _{d}} \big ( \lambda _{d}^{T} \otimes I_{n^{2}} \big ) \\ \end{bmatrix}y \\= & {} \displaystyle \bigg (\sum _{j=0}^{d} \mu _{j} \big ( \lambda _{j}^{T} \otimes I_{n^{2}} \big )^{T} \big ( \lambda _{j}^{T} \otimes I_{n^{2}} \big ) \bigg )y \\= & {} \displaystyle \big ((\sum _{j=0}^{d} \mu _{j} \lambda _{j}\lambda _{j}^{T}) \otimes I_{n^{2}} \big )y. \end{aligned}$$

Applying the orthogonality relation of the Krawtchouk polynomial (2.8), the (*r*, *s*)-th entry of $\sum _{j=0}^{d} \mu _{j} \lambda _{j}\lambda _{j}^{T}$ is $\sum _{j=0}^{d} \mu _{j}p_{r,j}p_{s,j} = q^{d}{d \atopwithdelims ()s}(q-1)^{s} \delta _{r,s} = q^{d}\mu _{s} \delta _{r,s}$ for $r,s = 0,\ldots ,d$. Thus ${{{\mathcal {T}}} } ({{{\mathcal {T}}} } ^*(y)) = {{\,\mathrm{{Diag}}\,}}(w)y$ and Assumption 4.1 is satisfied. $\square$

To efficiently solve the *Y*-subproblem for the **QAP** , we use Algorithm 4.3. Finally we describe how to obtain a valid lower bound when the **ADMM** model is solved approximately. The important problem of getting valid lower bounds from inaccurate solvers is recently discussed in [19].

##### Lemma 5.9

Let ${\mathcal {P}}$ be the feasible set defined in (5.15), and consider the problem in (5.17). For any ${\tilde{Z}} = ({\tilde{Z}}_{0},\ldots ,{\tilde{Z}}_{d})$, the objective value

5.22 $$\begin{aligned} g({\tilde{Z}}):= & {} \min \limits _{(Y_{0},\ldots ,Y_{d}) \in {\mathcal {P}}}\sum _{j=0}^{d} \langle {\tilde{C}}_{j} + {\tilde{Z}}_{j}, \sqrt{\mu _{j}}\sum _{i=0}^{d} p_{i,j}Y_{i} \rangle -\sum _{j=0}^{d}{\mu _{j}}p_{0,j} \lambda _{\max }({\tilde{V}}_{j}^{T}{\tilde{Z}}_{j}{\tilde{V}}_{j}) \nonumber \\\le & {} p^*, \end{aligned}$$

i.e., it provides a lower bound to the optimal value $p^{*}$ of (5.17).

##### Proof

The dual of (5.17) with respect to the constraints $\sqrt{\mu _{j}}(\sum _{i=0}^{d}p_{i,j}Y_{i} - {\tilde{V}}_{j}{\tilde{R}}_{j}{\tilde{V}}_{j}^{T}) = 0$ is

5.23 $$\begin{aligned} \begin{array}{ccl} d^{*}:=\max \limits _{({\tilde{Z}}_{0},\ldots ,{\tilde{Z}}_{d})}&\displaystyle \min \limits _{{\mathop {({\tilde{R}}_{0},\ldots ,{\tilde{R}}_{d}) \in {\mathcal {R}}}\limits ^{(Y_{0},\ldots ,Y_{d}) \in {\mathcal {P}}}}}&\displaystyle \sum _{j=0}^{d} \langle {\tilde{C}}_{j}, \sqrt{\mu _{j}}\sum _{i=0}^{d} p_{i,j}Y_{i} \rangle + \langle {\tilde{Z}}_{j}, \sqrt{\mu _{j}}(\sum _{i=0}^{d}p_{i,j}Y_{i} - {\tilde{V}}_{j}{\tilde{R}}_{j}{\tilde{V}}_{j}^{T}) \rangle . \end{array}\nonumber \\ \end{aligned}$$

The inner minimization problem can be written as

5.24 $$\begin{aligned} \begin{array}{c} \displaystyle \min \limits _{(Y_{0},\ldots ,Y_{d}) \in {\mathcal {P}}} \sum _{j=0}^{d} \langle {\tilde{C}}_{j} + {\tilde{Z}}_{j}, \sqrt{\mu _{j}}\sum _{i=0}^{d} p_{i,j}Y_{i} \rangle + \min \limits _{({\tilde{R}}_{0},\ldots ,{\tilde{R}}_{d}) \in {\mathcal {R}}} \sum _{j=0}^{d} \langle {\tilde{Z}}_{j}, \sqrt{\mu _{j}}( - {\tilde{V}}_{j}{\tilde{R}}_{j}{\tilde{V}}_{j}^{T}) \rangle . \end{array}\nonumber \\ \end{aligned}$$

It follows from the Rayleigh Principle, that the optimal value of the second minimization problem is $-\sum _{j=0}^{d}{\mu _{j}}p_{0,j} \lambda _{\max }({\tilde{V}}_{j}^{T}{\tilde{Z}}_{j}{\tilde{V}}_{j})$. Using strong duality, we have $g({\tilde{Z}}) \le d^{*} = p^{*}$. $\square$

#### Numerical results for the **QAP**

In this section we provide numerical results on solving the facially and symmetry reduced **DNN** relaxation (5.13). We first present our general stopping conditions and tolerances in Definition 5.10.

##### Definition 5.10

(tolerances, stopping conditions) Given a *tolerance parameter,* $\epsilon$, we terminate the **ADMM** when one of the following conditions is satisfied.

- The primal and dual residuals are smaller than $\epsilon$, i.e.,
  $$\begin{aligned} pres := \sum _{j=0}^{d}||\sum _{i=0}^{d}p_{i,j}Y_{i} - {\tilde{V}}_{j}{\tilde{R}}_{j}{\tilde{V}}_{j}^{T}|| < \epsilon \text { and } dres : = ||{{\tilde{Z}}}^{\text {old}} - {{\tilde{Z}}}^{\text {new}}|| \le \epsilon . \end{aligned}$$
- Let $p_{k}$ be the **ADMM** objective value, and $d_{k} := g({\tilde{Z}})$ the dual objective value at some dual feasible point at the *k*-th iteration, see (5.22). If the duality gap is not improving significantly, i.e.,
  $$\begin{aligned} {gap = \frac{p_{100k}-d_{100k}}{1+p_{100k}+d_{100k}}} < 10^{-4}, \end{aligned}$$
  for 20 consecutive integers *k*, then we conclude that there is stagnation in the objective value. We measure the gap only every 100-th iteration due to the expense of computing the dual objective value $d_{k}$.)

**Table 1:** Lower and upper bounds for different **QAP** instances

Problem	UB	MP [42]		**ADMM**			
		**LB**	Time	**OBJ**	**LB**	Time	res.
Harper_16	2752	2742	1	2743	2742	1.92	4.50e-05
Harper_32	27360	27328	3	27331	27327	9.70	1.67e-04
Harper_64	262260	262160	56	262196	261168	36.12	1.12e-05
Harper_128	2479944	2446944	1491	2446800	2437880	186.12	3.86e-05
Harper_256	22370940	–	–	22369996	22205236	432.10	9.58e-06
Harper_512	201329908	–	–	201327683	200198783	1903.66	9.49e-06
eng1_16	1.58049	1.5452	1	1.5741	1.5740	2.28	3.87e-05
eng1_32	1.58528	1.24196	4	1.5669	1.5637	14.63	5.32e-06
eng1_64	1.58297	0.926658	56	1.5444	1.5401	38.35	4.69e-06
eng1_128	1.56962	0.881738	1688	1.4983	1.4870	389.04	2.37e-06
eng1_256	1.57995	–	–	1.4820	1.3222	971.48	9.95e-06
eng1_512	1.53431	–	–	1.4553	1.3343	9220.13	9.66e-06
eng9_16	1.02017	0.930857	1	1.0014	1.0013	3.58	2.11e-06
eng9_32	1.40941	1.03724	3	1.3507	1.3490	12.67	3.80e-05
eng9_64	1.43201	0.887776	68	1.3534	1.3489	74.89	6.60e-05
eng9_128	1.43198	0.846574	2084	1.3331	1.3254	700.27	8.46e-06
eng9_256	1.45132	–	–	1.3152	1.2610	1752.72	9.74e-06
eng9_512	1.45914	–	–	1.3074	1.1168	23191.96	9.96e-06
VQ_32	297.29	294.49	3	296.3241	296.1351	11.82	1.27e-05
VQ_64	353.5	352.4	45	352.7621	351.4358	43.17	4.22e-04
VQ_128	399.09	393.29	2719	398.4269	396.2794	282.28	6.19e-04
rand_256	126630.6273	–	–	124589.4215	124469.2129	2054.61	3.78e-05
rand_512	577604.8759	–	–	570935.1468	569915.3034	9694.71	1.32e-04

In our **QAP** experiments, we use $\epsilon = 10^{-12}$ if $n \le 128$, and $\epsilon = 10^{-5}$ when $n=256,512$. The objective value from the **ADMM** is denoted by **OBJ** , and the valid lower bound obtained from the dual feasible solution is denoted by **LB** , see lemma 5.9. The running times in all tables are reported in seconds. We also list the maximum of the primal and dual residuals, i.e., *res* $:= \max \{pres,dres\}$. If a result is not available, we put *-* in the corresponding entry.

1. The first set of test instances are from Mittelmann and Peng [42], where the authors compute **SDP** bounds for the **QAP** with *A* being the Hamming distance matrix. Choices of the matrix *B*^9^ differ for different types of instances. In particular, in the Harper instance Harper_*n* where $n=2^d$ we have $B_{ij}=|i-j|$ for all $i,j=1,\ldots , 2^d$. Further eng1_*n* and end9_*n* with $n=2^d$, $d=4,\ldots , 9$ refer to the engineering problems, and VQ_*n* instances have random matrices *B*. For details see [42]. In rand_256 and rand_512 instances, *A* is the Hamming distance matrix of appropriate size and *B* is a random matrix. In the first column of Table 1 we list the instance names where the sizes of the **QAP** matrices are indicated after the underscore. Upper bounds are given in the column two. For instances with up to 128 nodes we list the upper bounds computed in [42], and for the remaining instances we use our heuristics. Since data matrices for the Harper instances are integer, we round up lower bounds to the closest integer. In the column three (resp. four) we list **SDP** -based lower bounds (resp. computation times in seconds) from [42]. The bounds from [42] are obtained by solving an **SDP** relaxation having several matrix variables on order *n*. The bounds in [42] were computed on a 2.67GHz Intel Core 2 computer with 4GB memory. In the columns five to seven, we present the results obtained by using our **ADMM** algorithm. Table 1 shows that we significantly improve bounds for all eng1_*n* and eng9_*n* instances. Moreover, we are able to compute bounds for huge **QAP** instances with $n=256$ and $n=512$ in a reasonable amount of time. Recall that for given *n*, the order of the matrix variable in the **DNN** relaxation of the **QAP**  (5.1) is $n^2$. However, for each instance $xx\_n$ of Table 1 we have that $n=2^d$, and that the **DNN** relaxation (5.1) boils down to $d+1$ positive semidefinite blocks of order *n*. In particular, we obtain the bound for each instance in Table 1 by solving the facially and symmetry reduced **DNN** relaxation (5.13) where ${\tilde{\mathbf{{\mathcal {B}}} }}^*_{k}(y) \in {\mathcal {S}}^{n}_+$, $k = 1,\ldots ,d+1$.
2. The second set of test instances are *Eschermann, Wunderlich, esc*, instances from the QAPLIB library [9]. In esc_*n*x instance, the distance matrix *A* is the Hamming distance matrix of order $n=2^d$, whose automorphism group is the automorphism group of the Hamming graph *H*(*d*, 2). In [15] the authors exploit symmetry in esc instances to solve the **DNN** relaxation (5.8) by the interior point method. That was the *first time* that **SDP** bounds for large QAP instances were computed by exploiting symmetry. In particular, the authors from [15] needed 13 seconds to compute the **SDP** bound for esc64a, and 140 seconds for computing the esc128 **SDP** bound, see also Table 2. The bounds in [15] are computed by the interior point solver SeDuMi [57] using the Yalmip interface [38] and Matlab 6.5, implemented on a PC with Pentium IV 3.4 GHz dual-core processor and 3GB of memory. Computational times in [15] include only solver time, not the time needed for Yalmip to construct the problem. In [44] the authors approximately solve the **DNN** relaxation (5.8) using the **ADMM** algorithm, but do note exploit symmetry. Here, we compare computational results from [44] with the approach we present in this paper. All the instances from [44] were tested on an Intel Xeon Gold 6130 2.10 Ghz PC with 32 cores and 64 GB of memory and running on 64-bit Ubuntu system. An efficient solver, called **SDPNAL**$+$, for solving large scale **SDPs** is presented in [59, 68]. **SDPNAL**$+$ implements an augmented Lagrangian based method. In particular, the implementation in [68] is based on a majorized semismooth Newton-CG augmented Lagrangian method, and the implementation in [59] is based on an inexact symmetric Gauss-Seidel based semi-proximal **ADMM** . In [59, 68], the authors present extensive numerical results that also include solving (5.1) on various instances from the QAPLIB library [9]. However, they do not perform **FR** and **SR**. In Table 2 we include results from [59] for solving esc_*n*x, with $n=16,32$. There are no results for $n=64, 125$ presented in their paper. Moreover the authors emphasize that **SDPNAL**$+$ is for solving **SDPs** where the maximum matrix dimension is assumed to be less than 5000. Due to the use of different computers, the times in Table 2 are not comparable. For example, the authors from [59] use an Intel Xeon CPU E5-2680v3, 2.50 GHz with 12 cores and 128 GB of memory. In Table 2 we present the numerical result for the esc instances. In particular, we compare bounds and computational times of the relaxation (5.1) (no reductions, solved in [59]), the facially reduced relaxation (5.6) (solved in [44]), the symmetry reduced relaxation (5.8) (solved in [15]), and facially and symmetry reduced relaxation (5.13) (solved by our **ADMM** ).

We conclude that:

1. There are notably large differences in computation times between the **ADMM**  algorithm presented here and the one from [44], since the latter does not exploit symmetry.
2. Even if the use of different computers is taken into account, this would likely not be enough to account for the time differences observed between our **ADMM** and **SDPNAL**$+$ [59]. Moreover, **SDPNAL**$+$ was not able to solve several instances.
3. In [15], the authors use SeDuMi to solve a relaxation equivalent to the symmetry reduced program (5.8); and they obtain a **LB** of 53.0844 for esc128. However, the bounds for this instance for the facially and symmetry reduced program (5.13) computed by the Mosek interior point method solver is 51.7516; and our **ADMM** algorithm reports 51.7518. This illustrates our improved numerical accuracy using **FR** and **SR**, and validates the statements about singularity degree, see Sect. 3.4. We note in addition that we provide a theoretically guaranteed lower bound, as well as solve huge instances that are intractable for the approach in [15].

**Table 2:** Esc instances (times with different computers)

inst.	opt	SDPNAL$+$ STYZ [59]		**ADMM** OWX [44]		**SDP** KS [15]		**ADMM**			
		**LB**	Time	**LB**	Time	**LB**	Time	**OBJ**	**LB**	Time	res
esc16a	68	63.2750	16	64	20.14	63.2756	0.75	63.2856	63.2856	2.48	1.17e-11
esc16b	292	289.9730	24	290	3.10	289.8817	1.04	290.0000	290.0000	0.78	9.95e-13
esc16c	160	153.9619	65	154	8.44	153.8242	1.78	154.0000	153.9999	2.11	2.56e-09
esc16d	16	13.0000	2	13	17.39	13.0000	0.89	13.0000	13.0000	1.04	9.94e-13
esc16e	28	26.3367	2	27	24.04	26.3368	0.51	26.3368	26.3368	1.21	9.89e-13
esc16f	0	–	–	0	3.22e+02	0	0.14	0	0	0.01	2.53e-14
esc16g	26	24.7388	4	25	33.54	24.7403	0.51	24.7403	24.7403	1.40	9.95e-13
esc16h	996	976.1857	10	977	4.01	976.2244	0.79	976.2293	976.2293	2.51	7.73e-13
esc16i	14	11.3749	6	12	100.79	11.3749	0.73	11.3749	11.3660	6.15	2.53e-06
esc16j	8	7.7938	4	8	56.90	7.7942	0.42	7.7942	7.7942	0.21	9.73e-13
esc32a	130	103.3206	333	104	2.89e+03	103.3194	114.88	103.3211	103.0465	12.36	3.62e-06
esc32b	168	131.8532	464	132	2.52e+03	131.8718	5.58	131.8843	131.8843	4.64	9.59e-13
esc32c	642	615.1600	331	616	4.48e+02	615.1400	3.70	615.1813	615.1813	8.04	2.05e-10
esc32d	200	190.2273	67	191	8.68e+02	190.2266	2.09	190.2271	190.2263	5.86	7.45e-08
esc32e	2	1.9001	149	2	1.81e+03	–	–	1.9000	1.9000	0.70	4.49e-13
esc32f	2	–	–	2	1.80e+03	–	–	1.9000	1.9000	0.76	4.49e-13
esc32g	6	5.8336	65	6	6.04e+02	5.8330	1.80	5.8333	5.8333	3.50	9.97e-13
esc32h	438	424.3256	1076	425	3.02e+03	424.3382	7.16	424.4027	424.3184	5.89	1.03e-06
esc64a	116	–	–	98	1.64e+04	97.7499	12.99	97.7500	97.7500	5.33	8.95e-13
esc128	64	–	–	–	–	53.0844	140.36	51.7518	51.7518	137.71	1.18e-12

### The graph partition problem (**GP**)

The graph partition problem is the problem of partitioning the vertex set of a graph into a fixed number of sets of given sizes such that the sum of edges joining different sets is minimized. The problem is known to be NP-hard. The **GP** has many applications such as VLSI design, parallel computing, network partitioning, and floor planing. Graph partitioning also plays a role in machine learning (see e.g., [34]) and data analysis (see e.g., [47]). There exist several **SDP** relaxations for the **GP** of different complexity and strength, see e.g., [30, 53, 54, 63, 67].

#### The general **GP** 

Let $G=(V,E)$ be an undirected graph with vertex set *V*, $|V|=n$ and edge set *E*, and $k\ge 2$ be a given integer. We denote by *A* the adjacency matrix of *G*. The goal is to find a partition of the vertex set into *k* (disjoint) subsets $S_1,\ldots , S_k$ of specified sizes $m_1\ge \ldots \ge m_k$, where $\sum _{j=1}^k m_j =n$, such that the sum of weights of edges joining different sets $S_j$ is minimized. Let

5.25 $$\begin{aligned} P_m := \left\{ S=(S_1,\ldots , S_k)\,|\, S_i\subset V, |S_i|=m_i, \forall i, ~~S_i\cap S_j = \emptyset , i \ne j, ~\cup _{i=1}^k S_i =V \right\} \nonumber \\ \end{aligned}$$

denote the set of all partitions of *V* for a given $m=(m_1,\ldots , m_k)$. In order to model the **GP** in binary variables we represent the partition $S\in P_m$ by the partition matrix $X\in {\mathbb {R}}^{n\times k}$ where the column *j* is the incidence vector for the set $S_j$.

The **GP** can be stated as follows

$$\begin{aligned} \min _{X\in {{\mathcal {M}}}_m} \frac{1}{2} {{\,\mathrm{{trace}}\,}}(AX(J_k-I_k)X^T), \end{aligned}$$

where

5.26 $$\begin{aligned} {{\mathcal {M}}}_m= \{ X\in \{0,1\}^{n\times k}\,|\, Xe_k= e_n, ~X^Te_n=m \} \end{aligned}$$

is the set of partition matrices.

Here, we consider the following **DNN** relaxation that is equivalent to the relaxation from [67]:

5.27 $$\begin{aligned} \begin{array}{cl} \min &{} \displaystyle \frac{1}{2} {{\,\mathrm{{trace}}\,}}( (A\otimes B) Y )\\ \text {s.t.} &{} {{\mathcal {G}}} (Y) = 0 \\ &{} {{\,\mathrm{{trace}}\,}}(D_{1} Y) - 2(e_{n} \otimes e_{k})^{T}{{\,\mathrm{{diag}}\,}}(Y) + n = 0 \\ &{} {{\,\mathrm{{trace}}\,}}(D_{2} Y) - 2(e_{n} \otimes m)^{T}{{\,\mathrm{{diag}}\,}}(Y) + m^{T}m = 0 \\ &{} {\mathcal {D}}_{O}(Y) = {{\,\mathrm{{Diag}}\,}}(m) \\ &{} {\mathcal {D}}_{e}(Y) = e \\ &{} \langle J ,Y \rangle = n^2 \\ &{} Y\ge 0, Y \succeq 0, \end{array} \end{aligned}$$

where $$B=J_k-I_k$$, and

$$\begin{aligned} Y = \begin{bmatrix} {Y}^{(11)} &{} \ldots &{} {Y}^{(1n)} \\ \vdots &{} \ddots &{} \vdots \\ {Y}^{(n1)} &{} \ldots &{} {Y}^{(nn)} \\ \end{bmatrix}\in {\mathcal {S}}^{kn} \end{aligned}$$

with each ${Y}^{(ij)}$ being a $k \times k$ matrix, and

$$\begin{aligned} \begin{array}{rl} D_{1} &{}= I_{n} \otimes J_k \\ D_{2} &{}= J_n \otimes I_{k} \\ {\mathcal {D}}_{O}(Y) &{}= \displaystyle \sum _{i=1}^{n}Y^{ii} \in {\mathcal {S}}^{k} \\ {\mathcal {D}}_{e}(Y) &{} = \displaystyle ({{\,\mathrm{{trace}}\,}}Y^{ii}) \in {\mathbb {R}}^{n} \\ {{\mathcal {G}}} (Y) &{}= \langle I_{n} \otimes (J_k - I_{k}) , Y \rangle . \end{array} \end{aligned}$$

Here ${{\mathcal {G}}} (\cdot )$ is the gangster operator for the **GP** . To compute **DNN** bounds for the **GP** , we apply **FR** for symmetry reduced relaxation (5.27). The details are similar to the **QAP** , and thus omitted.

**Table 3:** Graphs and partitions

Instance	|*V*|	# orbits	Blocks of *A*	*m*
1_FullIns_4	93	3629	(53,27,9,3,1)	(30,31,32)
can161	161	921	(20,20,20,20,20,20,20,11,10)	(52,53,56)
grid3dt5	125	4069	(39,36,26,24)	(40,41,44)
gridt15	120	2432	(80,24,16)	(39,40,41)
gridt17	153	3942	(102,30,21)	(50,51,52)
myciel7	191	6017	(64,64,63)	(62,63,66)

We present numerical results for different graphs from the literature. Matrix can161 is from the library Matrix Market [4], matrix grid3dt5 is 3*D* cubical mesh, and gridt*xx* matrices are 2*D* triangular meshes. Myciel7 is a graph based on the Mycielski transformation and 1_FullIns_4 graph is a generalization of the Mycielski graph. Both graphs are used in the COLOR02 symposium [28].

1. In Table 3 we provide information on the graphs and the considered 3-partition problems. In particular, the first column specifies graphs, the second column provides the number of vertices in a graph, the third column is the number of orbits after symmetrization, the fourth column lists the number of blocks in $Q^T AQ$. Here, the orthogonal matrix *Q* is computed by using the heuristic from [15]. The last column specifies sizes of partitions.
2. In Table 4 we list lower bounds obtained by using Mosek and our **ADMM** algorithm. The table also presents computational times required to compute bounds by both methods as well as the number of interior point method iterations. The results show that the **ADMM** with precision $\epsilon = 10^{-3}$ provides competitive bounds in much shorter time than the interior point method solver. In Table 4, some instances are marked by $*$. This means that our 64GB machine did not have enough memory to solve these instances by the interior point method solver, and therefore they are solved on a machine with an Intel(R) Xeon(R) Gold 6126, 2.6 GHz quad-core processor and 192GB of memory. However, the **ADMM** algorithm has much lower memory requirements, and thus the **ADMM** is able to solve all instances from Table 4 on the smaller machine.

**Table 4:** Numerical results for the graph 3-partition

Instance	**IPM** (Symmetry &Facially reduced)			**ADMM** $(\epsilon = 10^{-3})$			
	**LB**	Time	iter.	**OBJ**	**LB**	Time	res
1_FullIns_4	194.2825	311.95	26	194.2686	194.0523	141.29	1.50e-01
can161	33.0151	124.32	19	33.0392	30.4470	19.74	2.58e-01
grid3dt5	68.3175	245.65	17	68.3029	68.0436	200.35	2.02e-01
gridt15	12.1153	1302.10	41	12.1116	11.3654	97.17	1.91e-01
gridt17*	12.2482	1865.67	21	12.2532	11.1459	357.53	1.80e-01
myciel7*	1126.0309	2579.65	17	1126.0385	1123.8526	553.67	9.50e-02

#### The vertex separator problem (**VSP**) and min-cut (**MC**)

The min-cut problem is the problem of partitioning the vertex set of a graph into *k* subsets of given sizes such that the number of edges joining the first $k-1$ partition sets is minimized. The **MC** problem is a special case of the general **GP** but also arises as a subproblem of the vertex separator problem. The vertex separator problem is to find a subset of vertices (called vertex separator) whose removal disconnects the graph into $k-1$ components. This problem is NP-hard.

The vertex separator problem was studied by Helmberg et al. [25], Povh and Rendl [49], Rendl and Sotirov [51], Pong et al. [48]. The **VSP** appears in many different fields such as VLSI design [3] and bioinformatics [20]. Finding vertex separators of minimal size is an important problem in communication networks [33] and finite element methods [41]. The VSP also appears in divide-and-conquer algorithms for minimizing the work involved in solving systems of equations, see e.g., [36, 37].

Let us formally relate the **VSP** and the **MC** problem. Let $\delta (S_i,S_j)$ denote the set of edges between $S_i$ and $S_j$, where $S_i$ and $S_j$ are defined as in (5.25). We denote the set of edges with endpoints in distinct partition sets $S_1$,...,$S_{k-1}$ by

$$\begin{aligned} \delta (S) = \cup _{i<j<k} \delta (S_i,S_j). \end{aligned}$$

The min-cut problem is

$$\begin{aligned} \mathrm{cut}(m) = \min \{ |\delta (S)| \,|\, S\in P_m \}. \end{aligned}$$

The graph has a vertex separator if there exists $S\in P_m$ such that after the removal of $S_k$ the induced subgraph has no edges across $S_i$ and $S_j$ for $1\le 1<j<k$. Thus, if $\mathrm{cut}(m)=0$ or equivalently $\delta (S)=\emptyset$, there exists a vertex separator. On the other hand $\mathrm{cut}(m)> 0$ shows that no separator $S_k$ for the cardinalities specified in *m* exists.

Clearly, $|\delta (S)|$ can be represented in terms of a quadratic function of the partition matrix *X*, i.e., as $\frac{1}{2} {{\,\mathrm{{trace}}\,}}(AXBX^T)$ where

5.28 $$\begin{aligned} B := \begin{bmatrix} J_{k-1}-I_{k-1} &{}\quad 0 \\ 0 &{}\quad 0 \end{bmatrix} \in {\mathcal {S}}^{k}. \end{aligned}$$

Therefore,

$$\begin{aligned} \mathrm{cut}(m) = \min _{X\in {{\mathcal {M}}}_m} \frac{1}{2} {{\,\mathrm{{trace}}\,}}(AXBX^T), \end{aligned}$$

where ${{\mathcal {M}}}_m$ is given in (5.26). To compute **DNN** bounds for the **MC** problem and provide bounds for the vertex separator problem, we use the **DNN** relaxation (5.27) with *B* defined in (5.28).

1. We present numerical results for the Queen graphs, where the $n\times n$ Queen graph has the squares of an $n\times n$ chessboard for its vertices and two such vertices are adjacent if the corresponding squares are in the same row, column, or diagonal. The instances in this class come from the DIMACS challenge on graph coloring. In Table 5 we provide information on the Queen graphs. The table is arranged in the same way as Table 3.
2. In Table 6 we provide the numerical results for the vertex separator problem. More specifically, we are computing the largest integer $m_3$ such that the solution value of the **DNN** relaxation (5.27) is positive with partition
  5.29 $$\begin{aligned} m = \left( \left\lfloor \frac{n-m_3}{2} \right\rfloor , \lceil \frac{n-m_3}{2} \rceil , m_3\right) . \end{aligned}$$
  Then $m_3+1$ is a lower bound for the vertex separator problem with respect to the choice of *m*. One may tend to solve (5.27) for all possible $m_3$ between $0,1,\ldots ,|V|-1$ to find the largest $m_3$ for which the **DNN** bound is positive. However, the optimal value of (5.27) is monotone in $m_3$, and thus we find the appropriate $m_3$ using binary search starting with $m_3 = \lceil \frac{n}{2} \rceil$. We present the lower bound on the vertex separator, i.e., $m_3+1$ in the third column of Table 6. The total number of problems solved is listed in the fourth column of the same table. The running time given in the last two columns is the total amount of time used to find a positive lower bound for (5.27) for some $m_3$ by using Mosek and our **ADMM** algorithm, respectively. This task is particularly suitable for the **ADMM** , as we can terminate the **ADMM** once the lower bound in an iterate is positive. For example, it takes 786 seconds to solve the min-cut relaxation on queen12_12 by Mosek, see Table 7. However, though not shown in the table, it takes **ADMM** only 120 seconds to conclude that the optimal value is positive.
3. In Table 7 we compare bounds and computational times required to solve, for fixed *m*, symmetry reduced **DNN** relaxation (5.27) by the interior point algorithm, as well as symmetry and facially reduced relaxation (5.27) by using Mosek and our **ADMM** algorithm.

**Table 5:** The Queen graphs and partitions

instance	|*V*|	# orbits	blocks of *A*	*m*
queen5_5	25	91	(12,6,3,3,1)	(4,5,16)
queen6_6	36	171	(18,6,6,3,3)	(6,7,23)
queen7_7	49	325	(24,10,6,6,3)	(9,9,31)
queen8_8	64	528	(32,10,10,6,6)	(11,12,41)
queen9_9	81	861	(40,15,10,10,6)	(14,15,52)
queen10_10	100	1275	(50,15,15,10,10)	(18,18,64)
queen11_11	121	1891	(60,21,15,15,10)	(21,22,78)
queen12_12	144	2628	(72,21,21,15,15)	(25,26,93)
queen13_13	169	3655	(84,28,21,21,15)	(30,30,109)

**Table 6:** The vertex separator problem on the Queen graphs

instance	|*V*|	$m_3+1$	$\#$problems	**IPM** (Symmetry &Facially reduced) time	**ADMM** $(\epsilon = 10^{-12})$ time
queen 5_5	25	17	4	7.49	2.69
queen 6_6	36	24	5	9.62	2.91
queen 7_7	49	32	5	25.34	5.95
queen 8_8	64	42	6	85.72	34.35
queen 9_9	81	53	6	304.44	64.10
queen 10_10	100	65	7	1309.85	131.66
queen 11_11	121	79	7	3416.01	387.38
queen 12_12	144	94	7	6147.20	671.02
queen 13_13	169	110	8	–	1352.17

**Table 7:** The min-cut problem on the Queen graphs

	**IPM** (Symmetry reduced)			**IPM** (Symmetry &Facially reduced)			**ADMM** $(\epsilon = 10^{-12})$			
instance	**LB**	time	iter.	**LB**	time	iter.	**OBJ**	**LB**	time	res
queen5_5	0.0908	1.04	38	0.1658	0.27	10	0.1658	0.1658	6.88	7.36e-11
queen6_6	0.0962	3.43	31	0.1411	0.91	11	0.1411	0.1411	11.37	1.83e-10
queen7_7	0.5424	15.42	32	0.6196	1.92	10	0.6196	0.6196	17.97	5.53e-11
queen8_8	0.1967	127.60	39	0.3087	7.38	13	0.3087	0.3087	61.50	1.15e-10
queen9_9	0.0698	377.77	32	0.2175	19.98	12	0.2175	0.2175	204.39	1.16e-06
queen10_10	0.8159	1664.09	42	1.0211	85.42	14	1.0211	1.0211	239.75	1.09e-09
queen11_11	–	–	–	0.2131	275.20	16	0.2131	0.2131	877.85	1.82e-05
queen12_12	–	–	–	0.3248	786.12	25	0.3248	0.3248	1474.45	1.20e-06
queen13_13	–	–	–	–	–	–	0.9261	0.9261	1864.30	5.71e-09

We conclude from Tables 6 and 7 that

- For small instances, the interior point algorithm is faster than the **ADMM** as shown in Table 7. For larger instances, the interior point algorithm has memory issues. However, the **ADMM** algorithm can still handle large instances due to its low memory demand.
- To obtain bounds on the vertex separator of a graph, one does not need to solve the **DNN** relaxation to high-precision. The **ADMM** is able to exploit this fact, and find a lower bound on the size of the vertex separator in significantly less amount of time than the interior point algorithm, see Table 6.
- The symmetry reduced program without **FR** is heavily ill-conditioned, and the interior point method is not able to solve it correctly for any of the instances. The running time is also significantly longer than the symmetry and facially reduced program, see Tables 7. Note that we have solved the queen10_10 problem with high accuracy with **FR** . The distance between the optimal solutions in norm was very large with no decimals of accuracy. This emphasizes the importance of **FR** in obtaining accuracy in solutions, see e.g., [56].

## Conclusion

In this paper we propose a method to efficiently implement facial reduction to the symmetry reduced **SDP** relaxation, and we demonstrated the efficiency by solving large scale NP-hard problems. More specifically, if an exposing vector of the minimal face for the input **SDP** is given, then we are able to construct an exposing vector of the minimal face for the symmetry reduced **SDP**. The resulting relaxation is symmetry reduced, satisfies the Slater condition, and thus can be solved with improved numerical stability.

We then extend our reduction technique to doubly nonnegative, **DNN**, programs. In fact, our approach allows the matrix variable of the original **SDP**, to be passed to simple nonnegative vector for the **DNN**. Again we exploit exposing vectors of **DNN** as a decomposition into a sum of a semidefinite and nonnegative exposing vectors. Further, we discuss the importance of the order of the reductions in our theory. We also show that the singularity degree of the symmetry reduced program is equal to the singularity degree of the original program.

We apply our technique to many combinatorial problems and their **DNN** relaxations, i.e., we facially and symmetry reduce them. The obtained relaxations can be solved extremely efficiently using the alternating direction method of multipliers. We also show that interior point methods are more efficient on a symmetry and facially reduced relaxation. As a result, we are able to compute improved lower bounds for some **QAP** instances in significantly less time.

## Footnotes

1 Strong duality for the primal means a zero duality gap, $p^*_{{\textbf {SDP}}\,}=d^*_{{\textbf {SDP}}\,}$, and dual attainment.

2 Recall that a convex subset $F \subset C$ is a face of a convex set *C*, denoted $F \unlhd C$, if $x\in F, x\in {{\,\mathrm{{relint}}\,}}[y,z], y,z\in C \implies y,z\in F$. ere [*y*, *z*] is the line segment connecting *y* and *z*.

3 **FR** generally results in the constraints becoming linearly dependent. Therefore, a linearly independent subset need only be used [55].

4 The definition of an exposing vector follows that in (3.2). In this paper we assume, unless otherwise stated, that *W* exposes a face of a cone *K*, ${{\mathcal {F}}} \unlhd K$, means $W^\perp \cap K = {{\mathcal {F}}}$.

5 Our definition of singularity degree does not coincide with the definition from [58] when ${{\mathcal {P}}} _F=\{0\}$. In this case our definition gives ${{\,\mathrm{sd}\,}}({{\mathcal {P}}} _F)\ge 1$, while Sturm defines ${{\,\mathrm{sd}\,}}({{\mathcal {P}}} _F)=0$. See also [40] for discussions on the definition; and see [56] for lower bound results.

6 Note that this is always satisfied for basis matrices from a coherent configuration.

7 The link to the codes for the **QAP** can be found on the webpage (www.huhao.org). The codes for the other problems require finding symmetries in the graph; and therefore these codes and details can be obtained upon request directly from the authors.

8 We use ${{\mathcal {G}}}$ as the group and as a linear operator, usually referred to as the *gangster operator*, since the meaning is clear from the context. Here $\circ$ denotes the Hadamard product.

9 We thank Hans Mittelman for providing us generators for the mentioned instances.
